# Supplementary material for: SARS-CoV-2 spike N-terminal domain modulates TMPRSS2-dependent viral entry and fusogenicity
Source: Cell Rep. 2022 Aug 3;40(7):111220. doi: 10.1016/j.celrep.2022.111220 (PMC9346021; doi:10.1016/j.celrep.2022.111220)
Supplement: Document S1. Figures S1–S5 [file mmc1.pdf]

Cell Reports, Volume *40*

Supplemental information

SARS-CoV-2 spike N-terminal domain modulates TMPRSS2-dependent viral entry and fusogenicity

Bo Meng, Rawlings Datir, Jinwook Choi, CITIID-NIHR Bioresource COVID-19 Collaboration, John R. Bradley, Kenneth G.C. Smith, Joo Hyeon Lee, and Ravindra K. Gupta

Figure S1

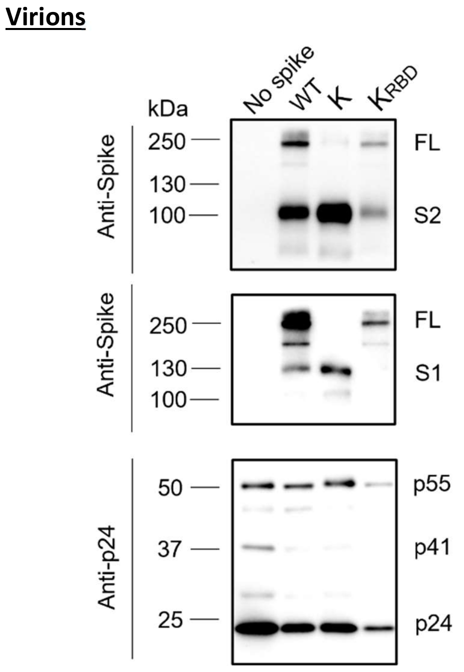

Supplementary Figure 1: Western blot of purified PV bearing RBD mutations only in SARS-CoV-2 Kappa and Kappa spike, together with WT and non-spike control. The sizes of protein markers were labelled to the left of the blot and the corresponding bands were labelled to the right. Related to Figure 1.

Figure S2

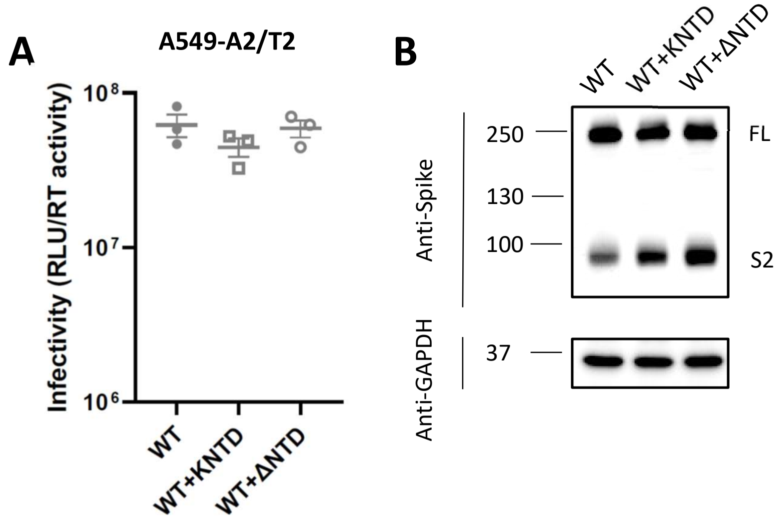

Supplementary Figure 2: The infectivity of SARS-CoV-2 WT, WT with Kappa NTD or Delta NTD chimeras in A549-A2/T2 cells (A) and western blot of 293T cell lysates showing spike cleavage (B). The sizes of protein markers were labelled to the left of the blot and the corresponding bands were labelled to the right. Related to Figure 1F.

Figure S3

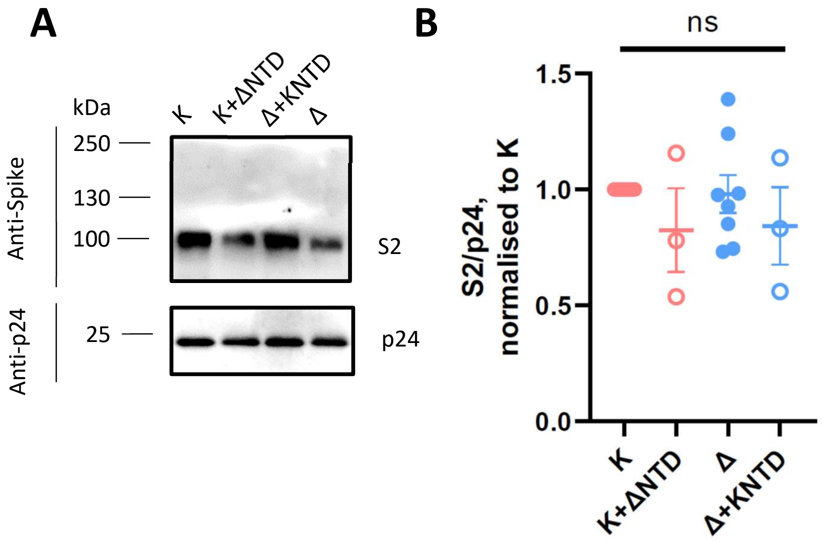

Supplementary Figure 3: (A): Western blots of purified PV bearing either SARS-CoV-2 Kappa, Delta or its chimeric spikes. The sizes of protein markers were labelled to the left of the blot and the corresponding bands were labelled to the right. (B): The intensity of the spike- and p24-associated bands on the western blots was densitometrically quantified (ImageJ) before the ratio was calculated for spike incorporation. Each dot represents one PV preparation and error bars represent SEM . The statistical analyses were done with one sample t test showing no significant difference. Related to Figure 2.

Figure S4

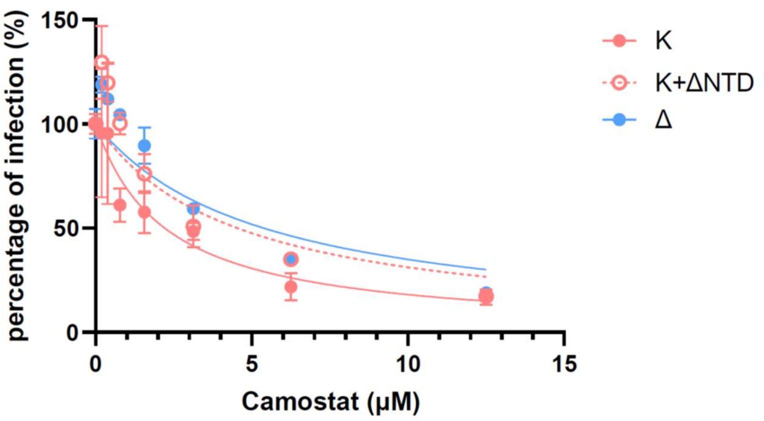

Supplementary Figure 4: The entry efficiency of SARS-CoV-2 Delta, Kappa and Kappa bearing the DeltaNTD in A549-ACE2/TMPRSS2 cells in the presence of TMPRSS2 specific inhibitor camostat. The RLU was normalised with non-drug control giving a percentage of infection. The data showing the SEM from 2 technical replicates; the error bars that lie within the datum points are not shown. Data are representative of two experiments. Related to Figure 2E.

Figure S5

A

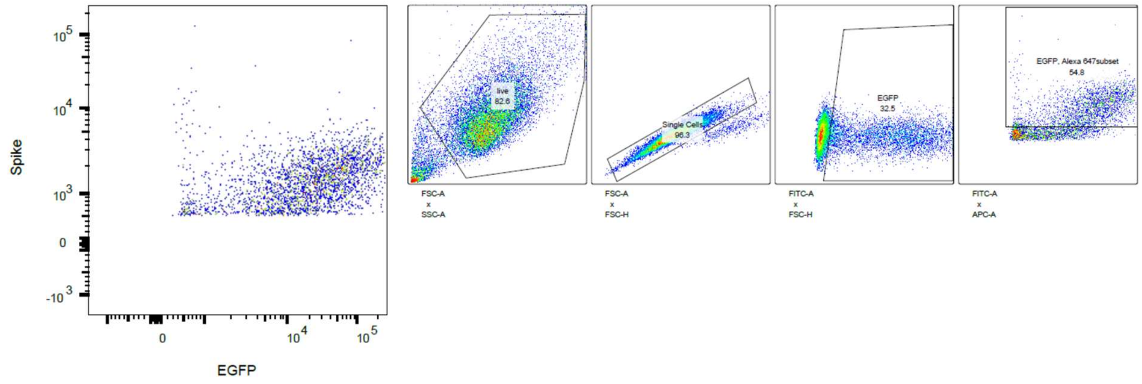

B

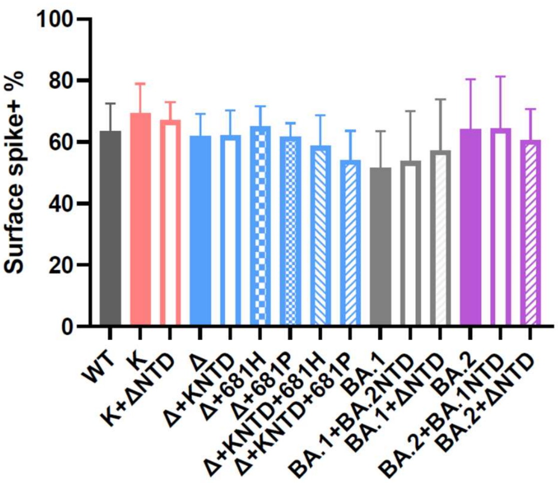

C

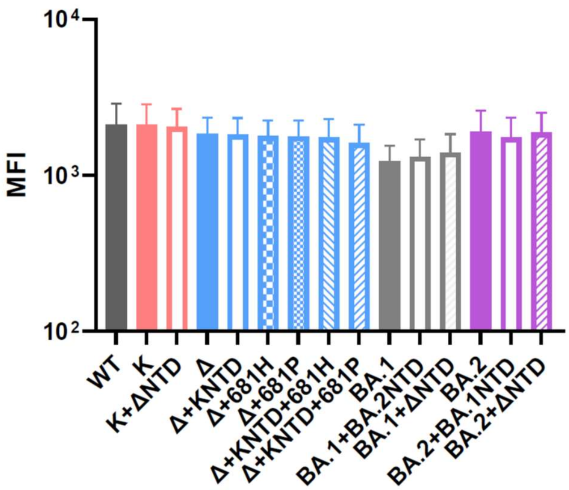

Supplementary Figure 5: (A): Gating strategy used to obtain the surface spike positive population in the transfected 293T cells. The spike positive cells from the eGFP positive cells were plotted either in percentage (B) or MFI (C) across different spikes. The error bars show the SEM from two experiments and the statistical analysis between each spike and WT was analysed with a paired t test, showing no significant difference. Related to Figures 4 and 5.
