# Supplementary material for: SARS-CoV-2 spike N-terminal domain modulates TMPRSS2-dependent viral entry and fusogenicity
Source: Cell Rep. 2022 Aug 3;40(7):111220. doi: 10.1016/j.celrep.2022.111220 (PMC9346021; doi:10.1016/j.celrep.2022.111220)
Supplement: Document S2. Article plus supplemental information [file mmc2.pdf]

Cell Reports

Article

SARS-CoV-2 spike N-terminal domain modulates TMPRSS2-dependent viral entry and fusogenicity

Graphical abstract

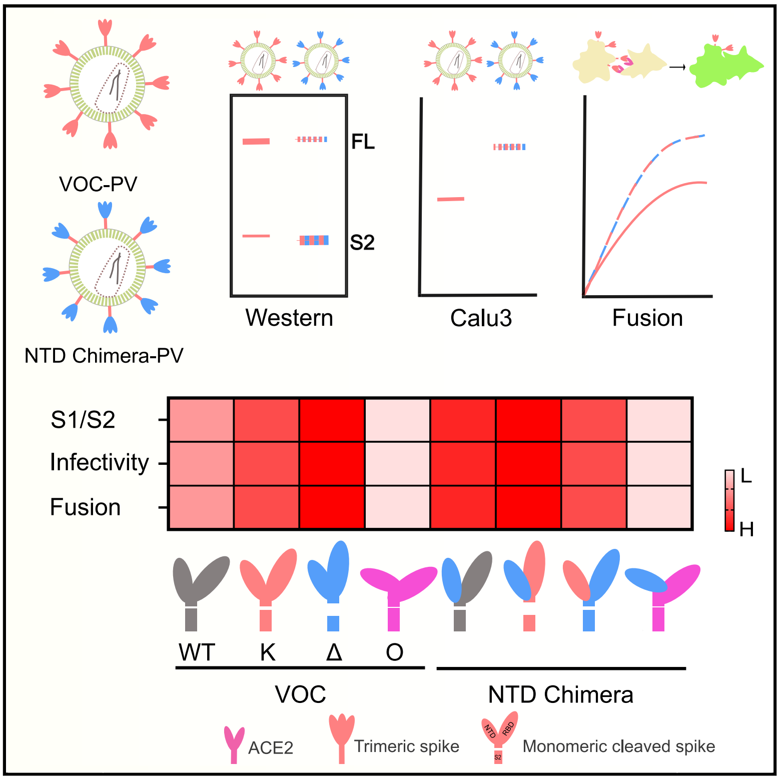

Highlights

- Delta NTD has enhanced S1/S2 cleavage in WT and Kappa, but not Omicron, through allostery
- S1/S2 cleavage efficiency correlates with TMPRSS2 usage for entry in Calu3 cells
- S1/S2 cleavage can be a proxy for prediction of efficiency in syncytium formation

Authors

Bo Meng, Rawlings Datir, Jinwook Choi, ..., Kenneth G.C. Smith, Joo Hyeon Lee, Ravindra K. Gupta

Correspondence

bm432@cam.ac.uk (B.M.), rkg20@cam.ac.uk (R.K.G.)

In brief

Meng et al. report that the NTD allosterically modulates S1/S2 cleavage and spike-mediated functions such as entry and cell-cell fusion in a spike context-dependent manner. These data may explain the lack of dominant SARS-CoV-2 inter-variant recombinants bearing breakpoints within spike.

Meng et al., 2022, Cell Reports *40*, 111220
August 16, 2022 © 2022 The Author(s).
https://doi.org/10.1016/j.celrep.2022.111220

CellPress

Cell Reports

CellPress
OPEN ACCESS

Article

SARS-CoV-2 spike N-terminal domain modulates TMPRSS2-dependent viral entry and fusogenicity

Bo Meng,[1,2,7,*] Rawlings Datir,[1,2] Jinwook Choi,[3] CITIID-NIHR Bioresource COVID-19 Collaboration, John R. Bradley,[2,4] Kenneth G.C. Smith,[1,2] Joo Hyeon Lee,[3,5] and Ravindra K. Gupta[1,2,6,*]

[1]Cambridge Institute of Therapeutic Immunology & Infectious Disease (CITIID), Cambridge, UK
[2]Department of Medicine, University of Cambridge, Cambridge, UK
[3]Wellcome-MRC Cambridge Stem Cell Institute, Cambridge, UK
[4]NIHR Bioresource, Cambridge, UK
[5]Department of Physiology, Development and Neuroscience, University of Cambridge, Cambridge, UK
[6]Africa Health Research Institute, Durban, South Africa
[7]Lead contact
*Correspondence: bm432@cam.ac.uk (B.M.), rkg20@cam.ac.uk (R.K.G.)
https://doi.org/10.1016/j.celrep.2022.111220

SUMMARY

The severe acute respiratory syndrome coronavirus 2 (SARS-CoV-2) spike N-terminal domain (NTD) remains poorly characterized despite enrichment of mutations in this region across variants of concern (VOCs). Here, we examine the contribution of the NTD to infection and cell-cell fusion by constructing chimeric spikes bearing B.1.617 lineage (Delta and Kappa variants) NTDs and generating spike pseudotyped lentivirus. We find that the Delta NTD on a Kappa or wild-type (WT) background increases S1/S2 cleavage efficiency and virus entry, specifically in lung cells and airway organoids, through use of TMPRSS2. Delta exhibits increased cell-cell fusogenicity that could be conferred to WT and Kappa spikes by Delta NTD transfer. However, chimeras of Omicron BA.1 and BA.2 spikes with a Delta NTD do not show more efficient TMPRSS2 use or fusogenicity. We conclude that the NTD allosterically modulates S1/S2 cleavage and spike-mediated functions in a spike context-dependent manner, and allosteric interactions may be lost when combining regions from more distantly related VOCs.

INTRODUCTION

The severe acute respiratory syndrome coronavirus 2 (SARS-CoV-2) furin cleavage site (also known as a polybasic cleavage site [PBSC]), cleaved in producer cells by a ubiquitously expressed protease furin (Hoffmann et al., 2020a), is believed to be one of the main reasons behind the success of SARS-CoV-2 worldwide (Johnson et al., 2021; Peacock et al., 2021). Upon virus release, the trimeric spike engages with angiotensin-converting enzyme 2 (ACE2) of the target cells to initiate virus entry (Jackson et al., 2022; Peng et al., 2021). Depending on the abundance of the cofactor TMPRSS2 and the status of spike cleavage (Hoffmann et al., 2020b; Ou et al., 2021; Park et al., 2016; Whittaker et al., 2021), the virus either enters through the TMPRSS2-mediated route by fusing at the plasma membrane or via late endosomes through a secondary cleavage preceding the concealed fusion peptide at S2' (Figure 1A) (Belouzard et al., 2009). The latter does not require a precleaved spike at S1/S2, as host proteases can mediate cleavage of both S1/S2 and S2' (Belouzard et al., 2009; Bosch et al., 2008; Jaimes et al., 2020; Park et al., 2016).

Kappa (B.1.617.1) and Delta (B.1.617.2) SARS-CoV-2 variants were first detected in India in late 2020 (Dhar et al., 2021; Ferreira et al., 2021). Although Kappa, the earliest detected B.1.617 variant in India, displayed greater escape to vaccine-elicited antibody responses (McCallum et al., 2021a), Delta surpassed Kappa to become the dominant strain in India and worldwide by mid-2021 (Mlcochova et al., 2021). The S gene encodes 8 and 9 non-synonymous mutations in Kappa and Delta, respectively, four of which are shared by both (Figure 1A). The N-terminal domain (NTD) is more variable between the two (3 and 4 mutations relative to Wu-1 in Kappa and Delta, respectively). In contrast, the receptor-binding domains (RBDs), each bearing two mutations, are more constrained, most likely due to their obligatory role in engaging with ACE2. Diverse mutations in the NTD are not unique to Kappa or Delta and have been documented in Alpha and many other variants of concern (VOCs) (McCarthy et al., 2021). Additionally, both Kappa and Delta share two conserved mutations at D614G and P681R and a unique mutation at S2, Q1071H for Kappa and D950N for Delta.

We previously showed that Kappa and Delta spikes exhibit highly efficient cleavage of S1/S2 over D614G wild type (WT) (Mlcochova et al., 2021). Intriguingly, Delta appears to be superior to Kappa in entering Calu3 cells and organoids expressing an endogenous level of ACE2 and TMPRSS2. Receptor binding is modestly increased in Delta but is lower than the preceding strains of VOCs, for example, Alpha (Collier et al., 2021a; Liu et al., 2022; Ulrich et al., 2022), indicating that enhanced receptor

This is an open access article under the CC BY license (http://creativecommons.org/licenses/by/4.0/).

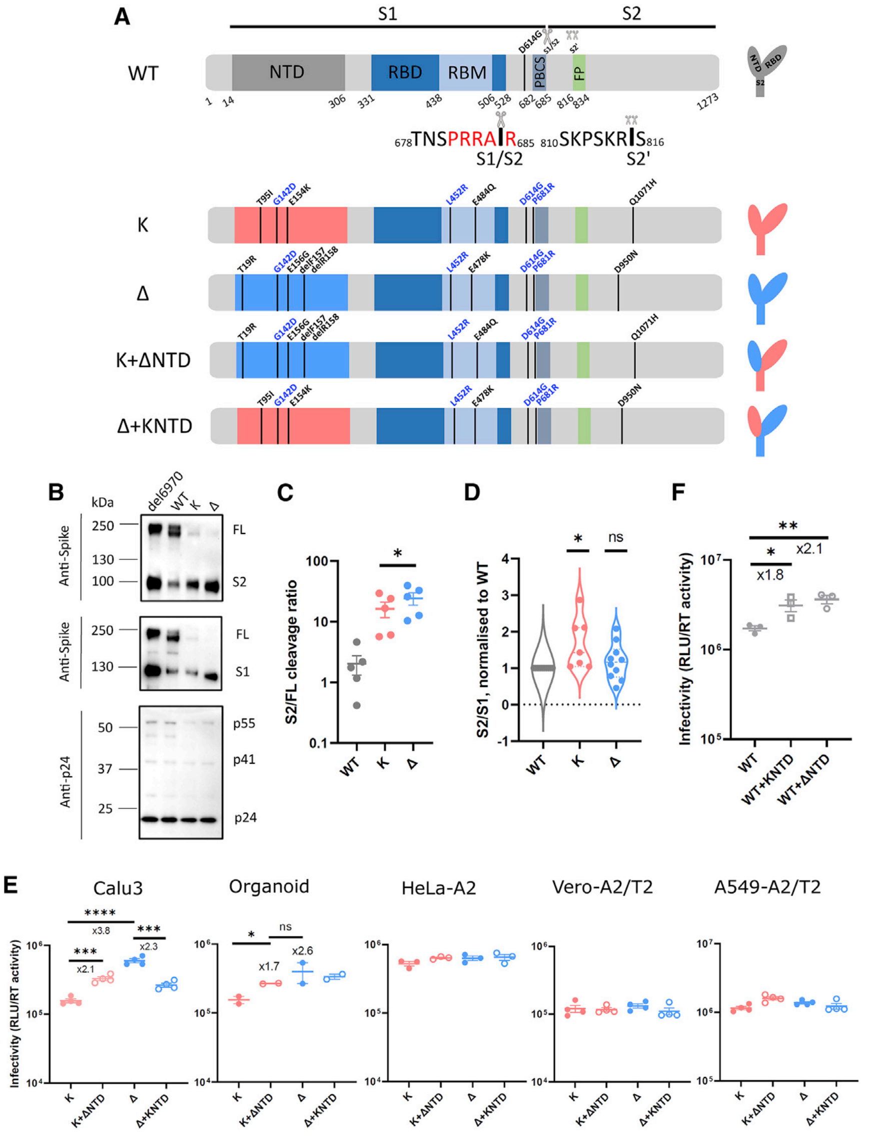

Figure 1. SARS-CoV-2 Delta exhibits increased infectivity over Kappa in Calu3 cells and is dependent on the NTD

(A) Schematic diagrams of WT (with D614G), Kappa, and Delta with their chimeras bearing swapped NTDs. The consensus mutations between Kappa and Delta are annotated in blue. The monomeric spikes shown on the right-hand side are for illustration purposes. PBCS, polybasic cleavage site; RBM, receptor-binding motif; FP, fusion peptide.

(B) Western blots of purified PVs bearing either H69V70 deletion or WT, Kappa, or Delta spikes. The sizes of protein markers are labeled to the left of the blot, and the corresponding bands are labeled to the right.

(C and D) The intensity of the spike-associated bands on the western blots was densitometrically quantified (ImageJ) before the ratio was calculated for cleavage (C; S2/FL, paired t test) or spike stability (D; S2/S1; one sample t test). In both (C) and (D), each dot represents one PV preparation.

(E) PV bearing Delta, Kappa, or chimeric spike was used to transduce Calu3 and organoids expressing endogenous levels of ACE2 and TMPRSS2 and ACE2/TMPRSS2-overexpressing cell lines including HeLa-ACE2, Vero-ACE2/TMPRSS2, and A549-ACE2/TMPRSS2. Unpaired t test.

(F) PV bearing WT, WT with Kappa NTD, and WT with Delta NTD were used to transduce Calu3 cells. In (E) and (F), mean ± SEM are shown for technical replicates (n = 2–4; two-sided unpaired Student t test). Data are representative of two to four experiments. ns, not significant, *$p < 0.05$, **$p < 0.01$, ***$p < 0.001$, ****$p < 0.0001$.

binding cannot solely explain the higher transmissibility of Delta (Barton et al., 2021; McCallum et al., 2021a; Ramanathan et al., 2021; Saville et al., 2022; Supasa et al., 2021). Moreover, cryoelectron microscopy (cryo-EM) structures of Delta and Kappa trimeric spikes show that both RBD and S2 adopt a remarkably similar geometry, suggesting that those two regions are less likely to be accountable for the increased entry of Delta over Kappa (Zhang et al., 2021a). *In vitro* studies using replication-competent virus isolates showed that Delta has fast replication kinetics in Calu3, human airway epithelium cells, and airway organoids (Mlcochova et al., 2021). However, the underlying molecular mechanism for the high transmissibility of Delta over Kappa in the real world is elusive.

Our published data also showed that the RBD on its own did not confer higher infectivity to Kappa (Ferreira et al., 2021), suggesting that the NTD may be responsible for the increased infectivity. The NTD interacts with cofactors L-SIGN and DC-SIGN at the cell surface (Lempp et al., 2021); blockade of these proteins can effectively neutralize the virus in ACE2 non-overexpressing cells, suggesting that NTD and RBD may work cooperatively. The cooperativity of the NTD and the RBD is additionally supported by the identification of infectivity-enhancing antibodies specifically targeting the NTD domain (Li et al., 2021; Liu et al., 2021) and the observation that binding of the 4A8 monoclonal antibody in the NTD modulates the RBD into an up position (Chi et al., 2020; Díaz-Salinas et al., 2022). Interestingly, such antibody-binding sites coincide with known infectivity enhancing sites, such as the H69V70 deletion that emerged during an example of intra-host evolution (Kemp et al., 2021) and in Alpha (Meng et al., 2021) and Omicron variants (Meng et al., 2022). It is therefore plausible that the NTD plays an active role in virus entry by engaging with host cofactors and triggering conformational changes of the RBD.

Despite there being over 20 mutations documented in the NTD, the role of those mutations in infectivity and their impact on the immune response elicited by vaccines is less clear. We reported that the H69V70 deletion found in Alpha was positively selected to increase its infectivity with a modest decrease in

immune evasion (Meng et al., 2021). Here, we hypothesized that the NTD plays a regulatory role that impacts S1/S2 cleavage and ACE2 usage. We constructed a panel of chimeric spike proteins with the NTDs from different VOCs in a variety of VOC backbones. We examined those chimeras alongside the parental VOCs in pseudovirus-based entry assays (Mlcochova et al., 2020) and investigated spike-mediated fusogenicity. Our data are consistent with a model whereby the NTD regulates virus entry and cell-cell fusion in a variant context-dependent manner.

RESULTS

NTD increases SARS-CoV-2 Delta infectivity in lung cells and airway organoids

The most dramatic changes in spike between Kappa and Delta lie in the NTD. Both Kappa and Delta spikes are efficiently cleaved in the producer cells (Mlcochova et al., 2021). We sought to assess the contribution of the NTD in spike cleavage in purified pseudotyped lentiviruses (PVs) by western blot. We included a deletion mutant in the NTD (delH69/V70) as a control due to its known efficient spike cleavage (Kemp et al., 2021; Meng et al., 2021). Plasmids encoding HIV Gag/pol, a genome flanked by long terminal repeats (LTRs) encoding luciferase, as well as the corresponding spike were transfected into 293T producer cells. The supernatants were harvested and pelleted through ultracentrifugation for western blot analysis. Our data show that the H69/V70 deletion increased S1/S2 cleavage compared with WT as expected (Figure 1B). Kappa and Delta spikes were efficiently cleaved, with a more pronounced cleavage observed in Delta (Figure 1C). We additionally observed that the Kappa spike was prone to S1 shedding, evidenced by a higher S2/S1 ratio compared with that of the WT (Figure 1D). In contrast, Delta spikes were more stable. We further noticed that the increased efficiency in spike cleavage requires a cognate NTD, as the spike bearing the RBD mutations alone failed to be cleaved efficiently (Figure S1), suggesting that the NTD on its own or together with the RBD influences the cleavage activity. We conclude that the Delta spike has evolved to be optimal in efficient spike cleavage while maintaining spike stability, reminiscent of the emergence of D614G in the early pandemic (Gobeil et al., 2021; Yurkovetskiy et al., 2020; Zhang et al., 2020, 2021b).

We previously observed enhanced cell-free infectivity for Delta over Kappa in Calu3 cells in the PV system (Mlcochova et al., 2021). Importantly, this difference was also evident for airway organoids (Mlcochova et al., 2021), suggesting that at endogenous expression levels of ACE2 and TMPRSS2, Delta has enhanced ability over Kappa in virus entry. Calu3 and airway organoids express high levels of TMPRSS2 (Meng et al., 2022). Given that cleavage efficiency at S1/S2 positively correlates with the infectivity in lung cells (Meng et al., 2022), we sought to examine whether the Delta NTD also contributes to higher infectivity over Kappa *in vitro*.

To test this, we constructed chimeras harboring either the Delta NTD or the Kappa NTD in Delta or Kappa backbones (Figure 1A). We then used the PV bearing different spikes to transduce cell lines to examine the entry efficiency. Interestingly, we observed elevated cell-free infectivity in a Kappa chimera bearing the Delta NTD in Calu3 cells and primary human airway organoids but not in other cell lines, indicating that the NTD confers the specificity for this increase (Figure 1E). Consistent with this model, a decrease in infectivity in Calu3 cells and primary human airway organoids was observed when the Kappa NTD was exchanged with that of Delta. We further extended this observation to the WT backbone, where a hybrid WT spike bearing either the Kappa or Delta NTD was expressed (Figures 1F and S2A). Consistent with these observations, both Kappa and Delta NTDs, when fused with WT, increased cell-free infectivity in Calu3 lung cells.

The NTD influences SARS-CoV-2 entry efficiency

We next sought to delineate the underlying mechanism for this increased infectivity in Delta over Kappa. Spike cleavage correlates with SARS-CoV-2 virus entry pathway preference (Meng et al., 2022; V'kovski et al., 2021). Western blots on purified pseudotyped virions showed a similar incorporation of spike for Delta, Kappa, and chimeras (Figure S3), suggesting the enrichment of spike at the cell surface is comparable. Analyses of the cell lysates from the 293T virus producer cells showed that the expression levels of chimeric spikes were also comparable (Figures 2A and 2B). However, the cleavage of spike at S1/S2 in the chimeras phenocopied the cleavage patterns from which their NTDs were derived (Figure 2C). We extended this observation in WT chimeras, where increased spike cleavage was also evident following addition of either the Kappa or Delta NTD (Figure S2B). Structural data indicate that the NTD, RBD, and PBCS are cooperatively regulated (Gobeil et al., 2021). Hence, we reasoned that the observed increase in the accessibility to the furin cleavage site may be regulated by the allostery involving the NTD.

SARS-CoV-2 primarily enters Calu3 cells through membrane fusion due to an abundant expression of TMPRSS2 and ACE2 at the plasma membrane. The primary cleavage at S1/S2 is a prerequisite for a secondary cleavage at S2′ site by proteases such as TMPRSS2 at the plasma membrane (V'kovski et al., 2021), which is closely correlated with the conformational rearrangement of S2 for the exposure of the fusion peptide. Due to more efficient cleavage of the Delta spike (Figure 1C), we reasoned that the Delta spike may be more efficiently primed to use TMPRSS2 at the plasma membrane for its entry. To test this hypothesis, we transduced the virus bearing either authentic spike of Delta or Kappa or their NTD-chimeric counterparts into TMPRSS2-overexpressing 293T cells or parental 293T cells (Figure 2D). As expected, all the viruses including chimeras increased entry efficiency when TMPRSS2 was present with an even more pronounced increase observed in Delta, consistent with the observation that Delta is more efficient in utilizing TMPRSS2. Intriguingly, we also observed an increased transduction efficiency for the Kappa chimera that was comparable to that of Delta, suggesting that the Delta NTD enables the entry of chimeric Kappa by efficient TMPRSS2 usage. Consistent with this model, the ratio of entry for the Delta chimera containing Kappa NTD entry in TMPRSS2-overexpressing cells versus normal-expressing cells was reduced.

To further investigate the dependence of TMPRSS2 on virus entry, we pretreated A549-ACE2/TMPRSS2 cells or airway organoids with either camostat (a TMPRSS2 inhibitor) or E64D (a cathepsin inhibitor). We observed that the half maximal inhibitory

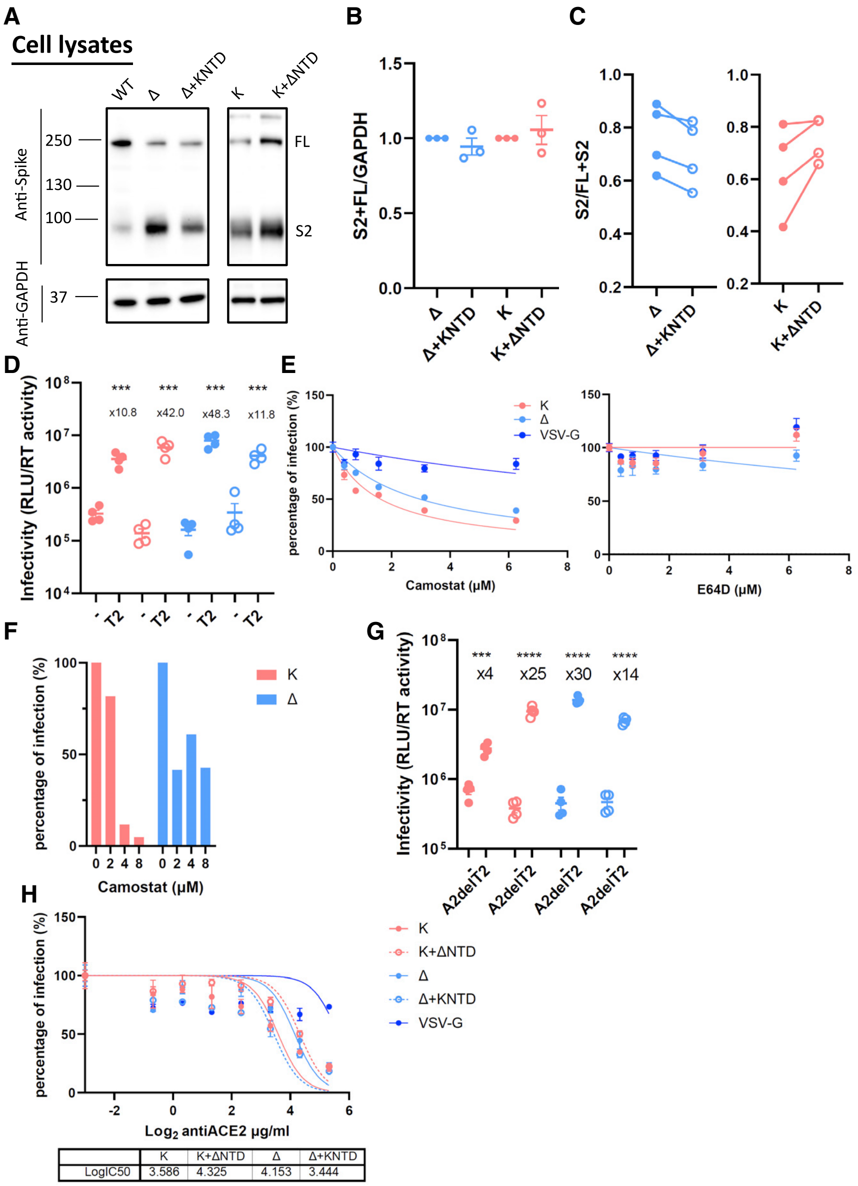

Figure 2. The NTD modulates the usage of TMPRSS2 and ACE2 for SARS-CoV-2 virus entry

(A) A representative western blot of transfected 293T cell lysates showing spike cleavage in Delta and Kappa and their chimeras. GAPDH was probed as a loading control.

(B) The band intensity from (A) was densitometrically calculated using ImageJ. Total S2-associated spike proteins (S2 and FL) were then normalized against GAPDH across Delta, Kappa, and NTD-bearing chimeras.

(C) The ratio of S2/FL+S2 from the band intensity of S2 and FL shown in (A) was plotted. $n \geq 3$.

(D) Delta, Kappa, or chimeras was transduced into either parental 293T cells or 293T cells overexpressing TMPRSS2. The fold increase of the virus entry in T2-overexpressing cells over parental cells is shown above the scatterplots. Mean ± SEM are shown for technical replicates (n = 4; two-sided unpaired Student's t test).

(E) The entry efficiency of Delta and Kappa in A549-ACE2/TMPRSS2 cells in the presence of TMPRSS2-specific inhibitor camostat or cathepsin-specific inhibitor E64D. The lentivirus pseudotyped with VSV-G was used as a control. The relative light unit (RLU) was normalized with non-drug-added control, giving a percentage of infection. The data showing the SEM from 4 experiments were plotted; the error bars that lie within the datum points are not shown.

(F) The entry efficiency of Delta and Kappa in the presence of camostat in airway organoids.

(G) Delta, Kappa, or chimeras was transduced into either parental 293T cells or 293T cells overexpressing ACE2 with abrogated TMPRSS2 expression (A2delT2). The fold increase of the virus entry in A2delT2 cells over parental cells is shown above the scatterplots. Mean ± SEM are shown for technical replicates (n = 4, two-sided unpaired Student's t test).

(H) 293T-A2delT2 cells were pretreated with anti-ACE2 antibody before the addition of PV bearing either Delta, Kappa, or chimeras together with virus pseudotyped with VSV-G. The data show the SEM from 2 technical replicates.

In (D) and (F)–(H), data are representative of two experiments. $^{***}p < 0.001$, $^{****}p < 0.0001$.

concentration (IC50) of camostat in Delta was approximately 2-fold higher than Kappa (1.6 μM for Kappa or 3.0 μM for Delta), while E46D had little effect on either virus (Figure 2E). Reassuringly, a similar observation was made in airway organoids (Figure 2F), suggesting that Delta is more resistant to camostat and hence is more efficient in utilizing available TMPRSS2 for virus entry. Indeed, the Kappa chimera bearing the Delta NTD showed a similar drug sensitivity profile to Delta, implying that the NTD is accountable for this shift in TMPRSS2 sensitivity (Figure S4).

It is reported that the NTD modulates RBD conformation through allosteric effects (Qing et al., 2021). We speculated that the accessibility to ACE2 is altered in the chimeric spikes. We next went on to examine this by transducing parental 293T cells or their isogenic counterparts where the expression of TMPRSS2 is abrogated and ACE2 is overexpressed (A2delT2). Indeed, we found a 30-fold increase in Delta when ACE2 is overexpressed, whereas Kappa only had 4-fold increase (Figure 2G). More strikingly, the Kappa chimera, bearing the Delta NTD in the Kappa spike backbone, showed a similar magnitude of increase as Delta. Conversely, the Delta chimera bearing a Kappa NTD manifested a decrease in ACE2 dependence compared with Delta, albeit to a lesser extent. To further examine the receptor usage, we pretreated 293T-A2delT2 cells with a titration series of anti-ACE2 antibody before being transduced with Delta, Kappa, or their chimeras. We observed that 50% more antibody was required to block the virus entry in Delta over Kappa (Figure 2H). It was evident that the efficient usage to ACE2 is dependent on the Delta NTD, as the chimeric Kappa bearing the authentic Delta NTD also increased its efficiency to ACE2

binding. Taken together, our data support the notion that the Delta NTD more efficiently modulates the use of TMPRSS2 due to spike cleavage and that the Delta NTD allosterically regulates the RBD to increase efficiency of ACE2 usage.

NTD mutations and deletions impact SARS-CoV-2 entry efficiency

To probe the mutations in the Delta NTD that may contribute to the enhanced infectivity in Calu3 cells, we constructed a series of spike bearing PVs by reverting a cluster of amino acids (142D, 156G, del157F, and del158R) to WT residues individually. Interestingly, each individually reverted mutant showed a decrease in infectivity in Calu3 cells, with the greatest reduction seen following re-insertion of the deleted 157R/158R (3-fold; Figure 3A). This is in agreement with the infectivity reduction in the Delta NTD bearing the Kappa chimera (Figure 1E). The decrease in infectivity was only observed in Calu3 cells but not in HeLa-ACE2 cells, consistent with the cell specificity in virus entry conferred by the NTD. Since the NTD harbors the antigenic supersites that are mutated in Delta (Cerutti et al., 2021; McCallum et al., 2021b; McCarthy et al., 2021; Suryadevara et al., 2021), we predicted that the reversion of such mutations would at least partially rescue the loss of sensitivity to the vaccine sera. To test this, PVs were harvested and used to transduce HeLa-ACE2 cells in the presence of a dilution series of vaccine sera from vaccinees who had received two doses of BNT162b2 at least 1 month prior to sampling (Figures 3B and 3C). D142G alone did not alter the sensitivity of neutralization (Figure 3C). However, G156E, or repairing the deletion of 157F/158R, increased the sensitivity of neutralization by 2-fold, consistent with the notion that the NTD is important for both immune evasion and infectivity.

SARS-CoV-2 delta NTD confers enhanced cell-cell fusion kinetics in a context-dependent manner

Syncytium formation, mediated by spike and the host receptor ACE2, has been found in SARS-CoV-2-infected patients and is thought to be important for disease progression (Braga et al., 2021). We and others previously demonstrated that variants bearing furin cleavage site mutations exhibit a pronounced increase in fusogenicity (Figure 4A), with the exception of Omicron (Buchrieser et al., 2021; Meng et al., 2021, 2022; Mlcochova et al., 2021; Rajah et al., 2021). While spike cleavage at S2′ is more closely correlated for fusion peptide (FP) exposure and fusion (Madu et al., 2009; Qing et al., 2022), we noticed that the cleavage at S1/S2 could serve as a reliable proxy for assessing the membrane fusion (Meng et al., 2021, 2022; Mlcochova et al., 2021). Given that we observed an enhanced cleavage at S1/S2 in the Kappa chimera, we sought to examine whether spike-mediated fusion is altered. We first confirmed that the surface spike at the plasma membrane is present at a similar level (Figure S5). We then went on to confirm that efficient cleavage is required for the syncytia formation in Delta and Delta bearing Kappa NTD, as the mutants bearing the WT proline at position 681 (681P) decreased fusion efficiency in both parental and chimeras, whereas the mutation to histidine (681H), the other cleavage site mutation firstly found in Alpha and then in Omicron, did not (Figure 4B). We then went on to explore whether or not the fusogenicity is different between Delta and Kappa and their chimeras. Concordant with the published studies, we found a marked increase in fusion for Delta and Kappa compared with WT (Figure 4C) (Mlcochova et al., 2021; Rajah et al., 2021). Unexpectedly, we found that the fusion kinetics between Delta and Kappa were noticeably different despite manifesting a comparably maximal fusion activity at the steady state (around 70%; Figure 4C). More specifically, Delta was more fusogenic at earlier time points, whereas Kappa required 2 more hours to reach a similar intensity. Intriguingly, when the Kappa NTD was fused into Delta, the fusion phenotype shifted from Delta to Kappa, with a slowing of kinetics (Figure 4C). Conversely, we observed a fast-fusing Delta phenotype when the Delta NTD was swapped into Kappa. Of note, we also observed a faster fusion phenotype when the Delta NTD was fused into the WT backbone, though this effect was less pronounced compared with that observed in the Kappa chimera (Figure 4C). Taken together, we have demonstrated that the Delta NTD can drive faster fusion in both WT and Kappa spike backgrounds.

Epidemiological studies suggest that the more recent Omicron (BA.1 and BA.2) variants are distantly related to all the other early pandemic variants (Simon-Loriere and Schwartz, 2022). It therefore remains possible that the ability of the SARS-CoV-2 Delta NTD to drive faster fusion is dependent on their spike background. We found that compared with BA.1, BA.2 was more efficient in entering Calu3 cells, though it was still not as efficient as Delta (Figure 5A). An elevated level of entry of BA.2 over BA.1 was observed in H1299 cells where the entry of Delta was the least efficient. These data suggest that BA.2 is capable of infecting both TMPRSS2-low (H1299) and -high (Calu3) cells efficiently.

We then introduced the Delta NTD into both BA.1 and BA.2 to examine whether the Delta NTD could also confer increased fusion to Omicron, a known poor cell-cell fusion spike (Figure 5B). Although BA.2 showed faster fusion than BA.1, consistent with a recently published study (Yamasoba et al., 2022), both Omicron and the Delta-NTD-fused Omicron chimeras showed poor spike-mediated fusion and that, most importantly, the difference between Omicron and its counterpart was negligible. Intriguingly, this phenotype correlates with inefficient cleavage of spike even after the cleavage-enhancing Delta NTD was fused into an Omicron spike background.

Omicron has evolved to avoid the early entry route via TMPRSS2 at the plasma membrane by entering the cells through endosomal route (Meng et al., 2022; Peacock et al., 2022; Willett et al., 2022) . Given that spike cleavage was not affected by domain swapping, we predicted that the Omicron chimera bearing the Delta NTD would fail to confer increased sensitivity to TMPRSS2, in contrast to our findings for the B.1.617 lineages Delta and Kappa (Figure 2D). Indeed, there was only a moderate increase in entry (2- to 3-fold) when TMPRSS2 was overexpressed, compared with Delta (9-fold), reinforcing the notion that the virus entry of Omicron is TMPRSS2 independent and that altering the NTD is not sufficient to confer TMPRSS2 usage to Omicron.

We next sought to examine whether the BA.2 NTD could rescue the poor fusogenicity of BA.1 and/or whether the BA.1 NTD impaired the fusogenicity of BA.2. In contrast to

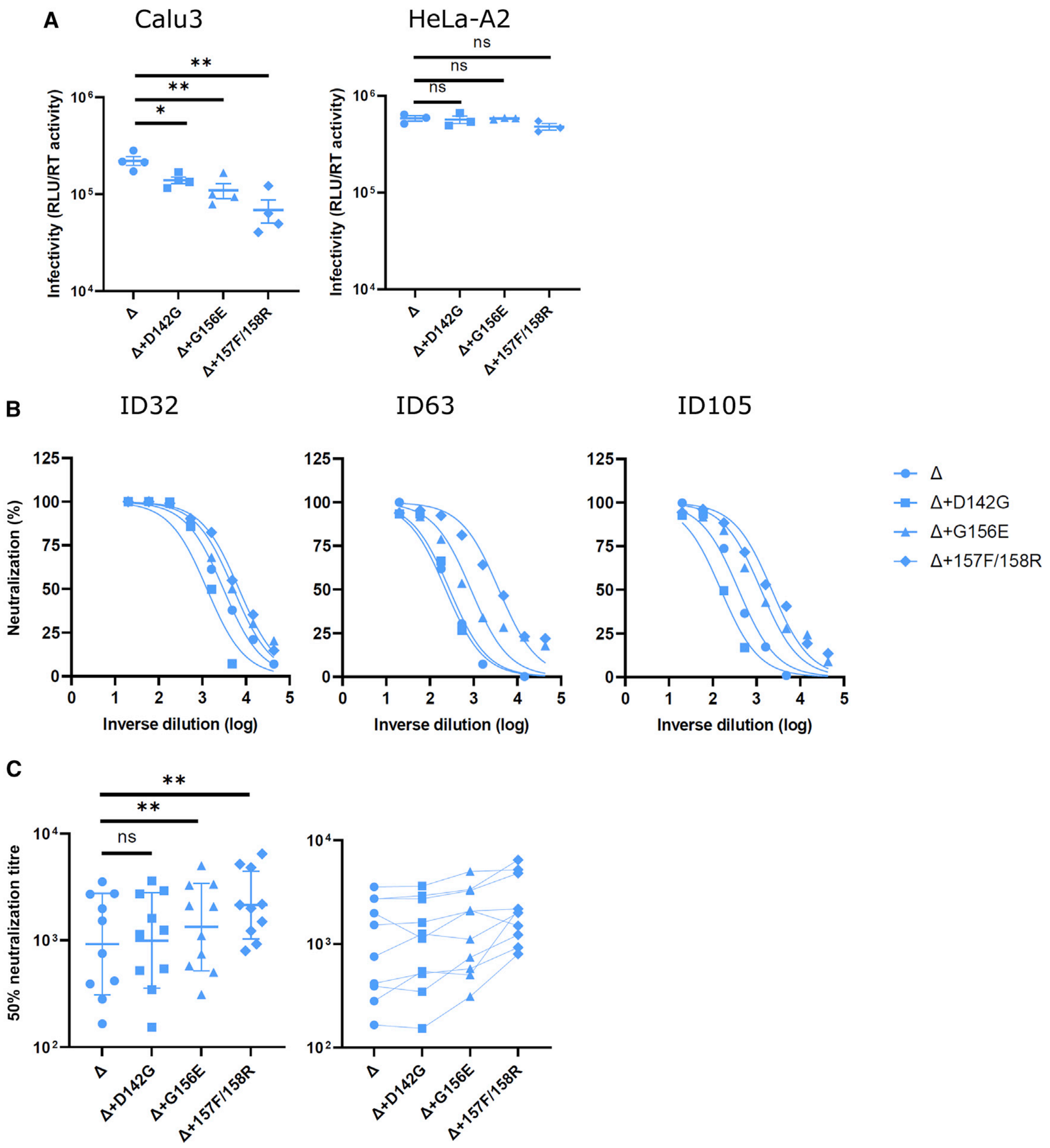

Figure 3. Reverting mutations in the Delta NTD toward WT reduces infectivity in lung cells and increases neutralization sensitivity to vaccine-elicited antibody

(A) PV bearing Delta and its reversions were used to transduce Calu3 and HeLa-ACE2 cells. Mean ± SEM are shown for technical replicates (n = 4; two-sided unpaired Student's t test).

(B) Examples of neutralization curves from ID32, -63, and -105 vaccinees with PVs bearing the reversion at 142, 156, or 157/8. Data points represent the mean of two technical replicates.

(C) The 50% serum neutralization was plotted across ten sera showing the geometric mean with geometric SD. Paired Wilcoxon was used for analysis. Data are representative of two experiments. ns, not significant, *$p < 0.05$, **$p < 0.01$.

the fast fusion activity conferred by the Delta NTD in Kappa or WT (Figure 4C), BA.2 NTD failed to increase the fusion kinetics of the BA.1 chimeric spike bearing the BA.2 NTD (Figure 5D). The BA.1 NTD modestly suppressed the fusion of BA.2 chimeric spike bearing the BA.1 NTD. Interestingly, when the sensitivity to TMPRSS2 was probed (Figure 5E), we found little difference between BA.1 and BA.2 and their chimeras (8- to 10-fold) compared with Delta (over 50-fold), indicating that in Omicron, the NTD on its own is not sufficient to affect TMPRSS2 utilization. Taken together, these data strongly suggest that the enhanced fusogenicity of the Delta NTD and the spike cleavage is context dependent and that non-NTD regions may be involved to suppress efficient fusion activity of Omicron spike.

CellPress
OPEN ACCESS

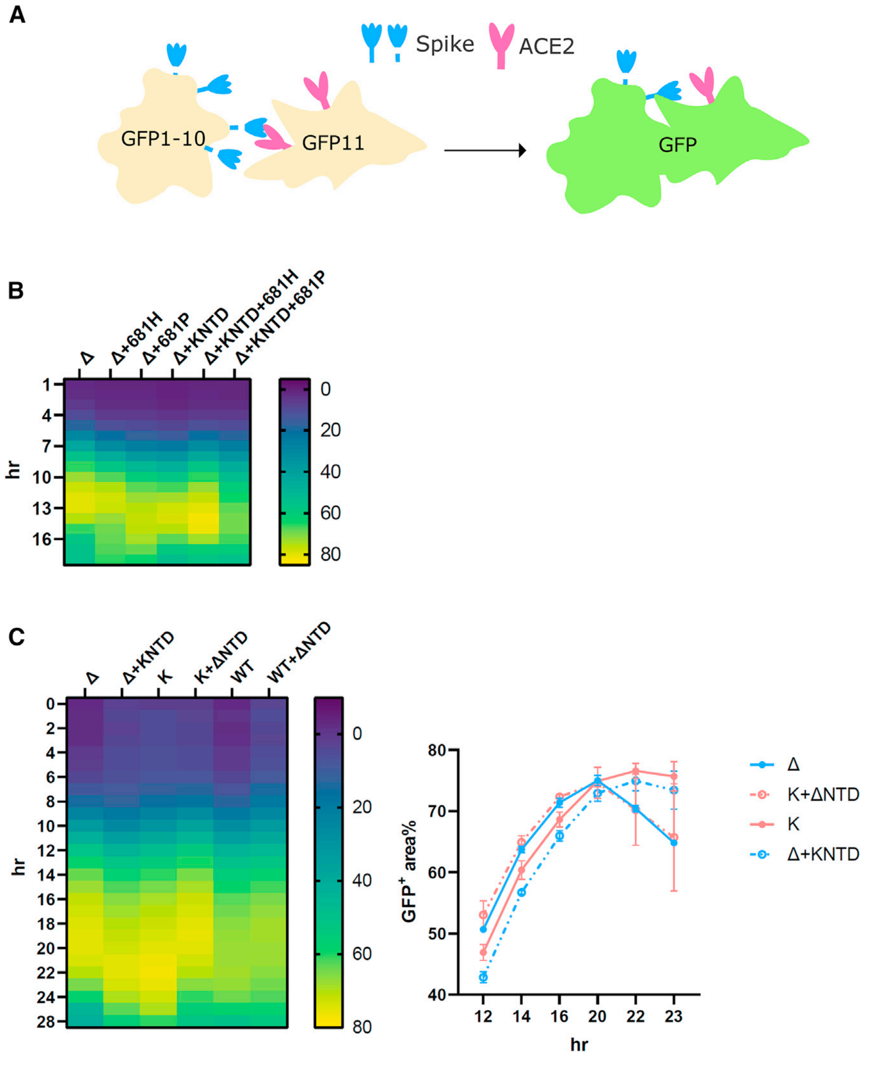

Figure 4. The SARS-CoV-2 Delta NTD increases fusion kinetics of Kappa and WT spikes

(A) A schematic diagram showing the split GFP system for spike-ACE2-mediated cell fusion.

(B) 681R or 681H is required for the enhanced fusogenicity in Delta and its chimera bearing the Kappa NTD.

(C) The fused Delta NTD in Kappa and WT increased the fusion kinetics of their counterparts, respectively. The line graphs on the right show the percentage of the positive GFP area at 12, 14, 16, 20, 22, and 23 h post transfection. The data showing the SEM at each time point were averaged from two experiments. The heatmap at each time point shows the mean of the GFP-positive area over the field of view from two experiments.

DISCUSSION

We previously showed that some escape mutations reduced virus entry efficiency, for example N439K and Y453F, and H69/V70 NTD deletion restored entry while maintaining immune evasion (Kemp et al., 2021; Meng et al., 2021). Therefore, it is possible that viruses have selected various mutations in the RBD due to the immune pressure imposed on this immunodominant site by the host. However, these RBD-mutated viruses may have suboptimal fitness, thus placing selection pressure in the other regions such as the NTD to acquire compensatory mutations. We propose that the NTDs in SARS-CoV-2 VOC have evolved under such pressure. Consistent with this notion, we observed that the Delta-NTD-revertant mutations (G156E and 157F/158R repair) were less efficient in entering lung cells and were less immune evasive (Figure 3). Reassuringly, a similar finding was recently reported in breakthrough B.1.617 viruses where the same mutations in the NTD accounted for the attenuated neutralization sensitivity (Mishra et al., 2022).

The NTD has been proposed to modulate the RBD to a receptor-accessible mode (Qing et al., 2021). We speculated that by alternating the NTD of Delta into Kappa that the receptor engagement with ACE2 is affected. Indeed, we observed an enhanced virus entry in the Kappa chimera bearing the Delta NTD (Figures 2G and 2H). However, an increased accessibility to ACE2 cannot solely account for the enhanced infectivity observed in Calu3, as such an increase is not evident for the cell lines where ACE2 is also abundantly expressed. A marked increase for chimeric Kappa spike to use TMPRSS2 at the plasma membrane provides a plausible explanation for this specificity; the chimeric Kappa spike is as efficient as Delta on TMPRSS2 usage (Figure 2D). This observation is in agreement with our inhibitor experiments, whereby the addition of the TMPRSS2 protease inhibitor camostat has a more pronounced inhibitory effect for Kappa than for Delta (Figures 2E and 2F), and fusing the Delta NTD in Kappa liberates the camostat inhibition to the level of Delta (Figure S4). However, the Delta NTD cannot enhance the cleavage of Omicron spike nor its dependence on TMPRSS2 (Figures 5B and 5C), suggesting that the presence of the Delta NTD is not the sole determinant of TMPRSS2 usage. Furthermore, although allosteric conformational changes involving NTD and other regions may contribute to phenotype (Raghuvamsi et al., 2021), binding of the NTD to a secondary cofactor at the plasma membrane cannot be completely excluded (McCallum et al., 2021b).

The NTD of VOCs, including poorly fusogenic Omicron (Meng et al., 2022; Suzuki et al., 2022), once fused with WT exhibits an increased fusion in the presence of trypsin, indicating the accessibility of spike cleavage by host protease is affected through the allostery of the NTD (Qing et al., 2022). We have proposed that the sensitivity to TMPRSS2 correlates with cleavage status at S1/S2, which has an additional impact on the cell-to-cell fusion

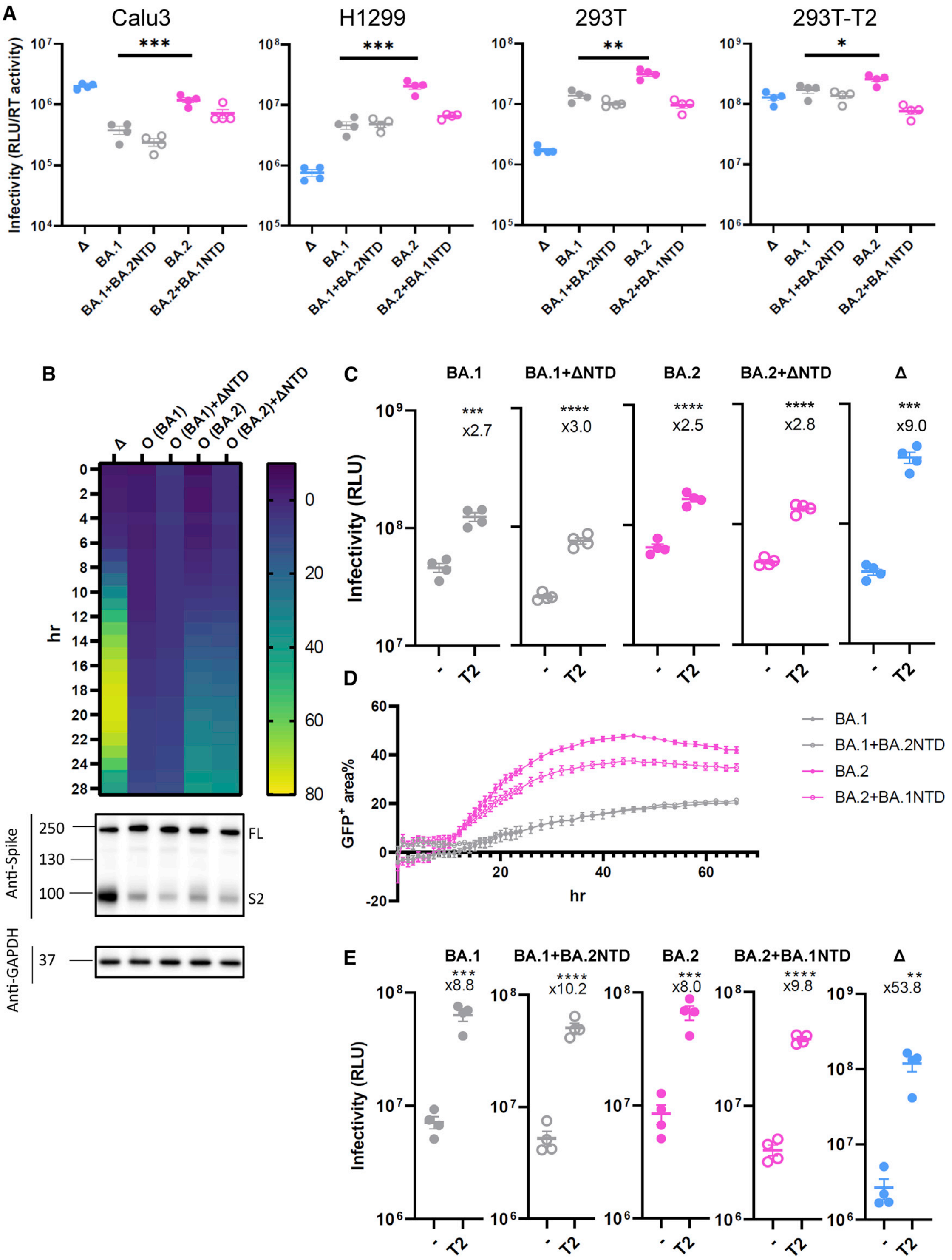

Figure 5. The SARS-CoV-2 Delta NTD or BA.2 NTD does not alter spike fusion or sensitivity to TMPRSS2 of BA.1

(A) PV bearing Delta, BA.1, BA.2, or chimeric forms of BA.1 and BA.2 spike were used to transduce Calu3, H1299, and 293T expressing endogenous levels of ACE2 and TMPRSS2 and TMPRSS2-overexpressing 293T cells.

(B) Fusion kinetics of the chimeric Delta NTD in BA.1 and BA.2 along with their parental spikes. The heatmap at each time point shows the mean of the GFP-positive area over the field of view from two experiments. The western blot showing cleavage of spike is directly underneath the heatmap.

(C) PV bearing BA.1, BA.2, or chimeras with Delta were transduced into either parental 293T cells or 293T cells overexpressing TMPRSS2. The fold increase of the virus entry in TMPRSS2-overexpressing cells over parental cells is shown above the scatterplots.

(legend continued on next page)

(Meng et al., 2022). Consistent with this notion, we found the Delta NTD confers faster fusion kinetics in both WT and Kappa, which coincides with an elevated S1/S2 cleavage in WT and Kappa spike bearing the Delta NTD. However, S1/S2 cleavage is unaltered in the Delta NTD bearing Omicron, which also has poor fusogenicity (Figure 5B). These data demonstrated that regions other than the NTD may also be required for efficient cleavage. Indeed, a recent study using a domain-swapping approach has suggested that the RBD of Omicron contributes to inefficient spike cleavage and inferior fusogenicity (Kimura et al., 2022). The lack of allostery of the Omicron NTD may reside in the unique nature of its spike (Cerutti et al., 2022; Cui et al., 2022; Gobeil et al., 2022; Mannar et al., 2022; McCallum et al., 2022; Stalls et al., 2022; Ye et al., 2022; Zhang et al., 2022). Compared with the highly dynamic Delta spike, the Omicron spike is more packed due to the newly acquired inter- and intra-domain interactions (Gobeil et al., 2022; Zhang et al., 2022). Firstly, the tighter inter-protomeric couplings are facilitated by the new substitutions S373P and S375F from one protomer, with N501Y and Y505H on the other, in the all 3-RBD-down position or between S375F and F486 in the 1-RBD-up structure (Cui et al., 2022; Gobeil et al., 2022; Zhou et al., 2022). Secondly, intra-domain interactions within the S1 region have also increased the inflexibility of otherwise highly mobile features such as the NTD and NTD to RBD (N2R) in S1 (Gobeil et al., 2022). Thirdly, the FP proximal region (FPPR) and 630 loop are more visible in the 1-RBD-up position (Cui et al., 2022; Zhang et al., 2022), suggesting that these two rigidified structural features could increase the energy level that is required to bolster more RBD to the up position, which may in turn impair the spike cleavage. The inter-domain interactions between two β strands in close proximity of S2′ cleavage site that was proposed to coordinate the NTD-induced spike cleavage (Qing et al., 2022) may also be impaired in Omicron. Taking together the structural data, it is plausible that the uniqueness of the aforementioned structural features in Omicron may contribute to the incompatibility when the NTD from other strains was fused into the Omicron backbone.

We observed that the Kappa spike is less stable than that of Delta (Figures 1B and 1D). We suspect that when Delta first emerged with the RBD mutations, the intermediate strain was less fit, leading to a premature S1 shedding. However, the subsequently acquired mutations in the NTD allosterically foster non-covalent interactions between S1 and S2, forming more stabilized protomers before engagement with ACE2. This process ultimately leads to active S1 shedding and exposure of the FP (Raghuvamsi et al., 2021). D614G emerged as a positively selected mutation in an early stage of the pandemic, persisting in subsequent lineages by stabilizing trimeric spike. The more stable form of S1 is therefore better positioned to engage with ACE2 (Daniloski et al., 2021; Díaz-Salinas et al., 2022; Yang et al., 2022; Yurkovetskiy et al., 2020; Zhang et al., 2020, 2021b). It is reasonable to speculate that the effect of the NTD mutations is analogous to the acquisition of D614G by strengthening the intra-molecular interactions to prevent S1 from being loosened prematurely while bolstering the RBD in an ACE2-accessible form.

In summary, our data support the cooperativity between NTD, RBD, and the furin cleavage site. We propose that the NTD allosterically affects the conformation of the RBD for ACE2 binding and the furin cleavage site for spike cleavage. The precleaved spike confers specificity on virus entry via TMPRSS2 usage and drives a faster virus spread with more efficient spike-mediated fusion. Our model explains the dominance of Delta over Kappa by being highly immune evasive on the one hand through the acquisition of known escape mutations, while on the other hand gaining S1/S2 stability and increased infectivity in lung cells. Our study highlights the importance of the continuous effort in monitoring both RBD and NTD mutations in order to understand the biology of variants and possibly explains the lack of dominant SARS-CoV-2 inter-variant recombinants bearing breakpoints within spike. On the translational/therapeutic side, combination antibody therapy targeting both the NTD and the RBD might therefore be less prone to resistance than monotherapy or combinations targeting the RBD alone.

Limitations of the study

Our study was based on viruses pseudotyped with different spikes of interest. Although significant consistency has been observed between the PV system and the live-virus system regarding SARS-CoV-2 entry, there is still a possibility that the observation made in PV might be different compared with the real live-virus system. Hence, chimeric NTD infectious clones are highly desirable to confirm our observations.

STAR★METHODS

Detailed methods are provided in the online version of this paper and include the following:

- KEY RESOURCES TABLE
- RESOURCE AVAILABILITY
  - Lead contact
  - Materials availability
  - Data and code availability
- EXPERIMENTAL MODEL AND SUBJECT DETAILS
  - Ethical approval
  - Cell culture
- METHOD DETAILS
  - Plasmids
  - Pseudotype virus preparation and infectivity titration
  - PV SG-PERT
  - Western blot

(D) The fusion assay of BA.1, BA.2, and their chimeric BA.1 bearing the BA.2 NTD and BA.2 bearing the BA.1 NTD. The line graphs show the percentage of the positive GFP area at 1 h interval post transfection. The data showing the SEM at each time point were averaged from two experiments.
(E) BA.1, BA.2, or chimeras bearing the BA.2 NTD or the BA.1 NTD together with Delta were transduced into either parental 293T cells or 293T cells overexpressing TMPRSS2. The fold increase of the virus entry in T2-overexpressing cells over parental cells is shown above the scatterplots.
In (A), (C), and (E), the plots are representative of two experiments. Mean ± SEM are shown for technical replicates (n = 4; two-sided unpaired Student's t test). $^{*}p < 0.05$, $^{**}p < 0.01$, $^{***}p < 0.001$, $^{****}p < 0.0001$.

- Drug and receptor blocking assay
- Cell-cell fusion assay
- FACS for surface spike expression
- Neutralisation assay
- QUANTIFICATION AND STATISTICAL ANALYSIS

SUPPLEMENTAL INFORMATION

Supplemental information can be found online at https://doi.org/10.1016/j.celrep.2022.111220.

CONSORTIA

Stephen Baker, Gordon Dougan, Christoph Hess, Nathalie Kingston, Paul J. Lehner, Paul A. Lyons, Nicholas J. Matheson, Willem H. Owehand, Caroline Saunders, Charlotte Summers, James E.D. Thaventhiran, Mark Toshner, Michael P. Weekes, Patrick Maxwell, Ashley Shaw, Ashlea Bucke, Jo Calder, Laura Canna, Jason Domingo , Anne Elmer, Stewart Fuller, Julie Harris, Sarah Hewitt, Jane Kennet, Sherly Jose, Jenny Kourampa, Anne Meadows, Criona O'Brien, Jane Price, Cherry Publico, Rebecca Rastall, Carla Ribeiro, Jane Rowlands, Valentina Ruffolo, Hugo Tordesillas, Ben Bullman, Benjamin J. Dunmore, Stuart Fawke, Stefan Gräf , Josh Hodgson, Christopher Huang, Kelvin Hunter, Emma Jones, Ekaterina Legchenko, Cecilia Matara, Jennifer Martin, Federica Mescia, Ciara O'Donnell, Linda Pointon, Joy Shih, Rachel Sutcliffe, Tobias Tilly, Carmen Treacy, Zhen Tong, Jennifer Wood, Marta Wylot, Ariana Betancourt, Georgie Bower, Chiara Cossetti, Aloka De Sa, Madeline Epping, Stuart Fawke, Nick Gleadall, Richard Grenfell, Andrew Hinch, Sarah Jackson, Isobel Jarvis, Ben Krishna, Francesca Nice, Ommar Omarjee, Marianne Perera, Martin Potts, Nathan Richoz, Veronika Romashova, Luca Stefanucci, Mateusz Strezlecki, Lori Turner, Eckart M.D.D. De Bie, Katherine Bunclark, Masa Josipovic, Michael Mackay, John Allison, Helen Butcher, Daniela Caputo, Debbie Clapham-Riley, Eleanor Dewhurst, Anita Furlong, Barbara Graves, Jennifer Gray, Tasmin Ivers, Emma Le Gresley, Rachel Linger, Sarah Meloy, Francesca Muldoon, Nigel Ovington, Sofia Papadia, Isabel Phelan, Hannah Stark, Kathleen E Stirrups, Paul Townsend, Neil Walker, Jennifer Webster, Ingrid Scholtes, Sabine Hein, Rebecca King

ACKNOWLEDGMENTS

R.K.G. is supported by a Wellcome Trust Senior Fellowship in Clinical Science (WT108082AIA). This study was supported by the Cambridge NIHR Biomedical Research Centre, Addenbrooke's Charitable Trust, and the Rosetrees Trust. We would like to thank Paul Lehner for Calu3, James Voss for HeLa ACE2, Simon Cook for H1299, and Suzanne Rihn for the A549 cells. We are grateful to Leo James for 293T-ACE2-ΔTMPRSS2, 293T-TMPRSS2, Vero-GFP1-10, and 293T-GFP11 cells and to Guido Papa and Anna Albecka for the help on imaging. The authors acknowledge plasmids from the G2P-UK National Virology consortium funded by MRC/UKRI (grant ref. MR/W005611/1) and thank Tom Peacock and Wendy Barclay. We acknowledge the help from the Cambridge NIHR Biomedical Research Center Cell Phenotyping Hub and the NIHR Bioresource. The views expressed are those of the author(s) and not necessarily those of the NIHR.

AUTHOR CONTRIBUTIONS

B.M. and R.D. performed experiments. B.M. and R.K.G. conceived the study. B.M., R.K.G., J.C., J.R.B., K.G.C.S., and J.H.L. planned the experiments and interpreted the data. B.M. wrote the initial draft of the manuscript. All authors reviewed and approved the manuscript.

DECLARATION OF INTERESTS

R.K.G. has received honoraria for consulting and educational activities from Gilead, GSK, Janssen, and Moderna. All the other authors declare no competing interests.

Received: May 6, 2022
Revised: June 30, 2022
Accepted: July 22, 2022
Published: August 3, 2022

REFERENCES

Barton, M.I., MacGowan, S.A., Kutuzov, M.A., Dushek, O., Barton, G.J., and van der Merwe, P.A. (2021). Effects of common mutations in the SARS-CoV-2 Spike RBD and its ligand, the human ACE2 receptor on binding affinity and kinetics. Elife *10*, e70658. https://doi.org/10.7554/eLife.70658.

Belouzard, S., Chu, V.C., and Whittaker, G.R. (2009). Activation of the SARS coronavirus spike protein via sequential proteolytic cleavage at two distinct sites. Proc. Natl. Acad. Sci. USA *106*, 5871–5876. https://doi.org/10.1073/pnas.0809524106.

Bosch, B.J., Bartelink, W., and Rottier, P.J.M. (2008). Cathepsin L functionally cleaves the severe acute respiratory syndrome coronavirus class I fusion protein upstream of rather than adjacent to the fusion peptide. J. Virol. *82*, 8887–8890. https://doi.org/10.1128/JVI.00415-08.

Braga, L., Ali, H., Secco, I., Chiavacci, E., Neves, G., Goldhill, D., Penn, R., Jimenez-Guardeño, J.M., Ortega-Prieto, A.M., Bussani, R., et al. (2021). Drugs that inhibit TMEM16 proteins block SARS-CoV-2 spike-induced syncytia. Nature *594*, 88–93. https://doi.org/10.1038/s41586-021-03491-6.

Buchrieser, J., Dufloo, J., Hubert, M., Monel, B., Planas, D., Rajah, M.M., Planchais, C., Porrot, F., Guivel-Benhassine, F., van der Werf, S., et al. (2021). Syncytia formation by SARS-CoV-2-infected cells. EMBO. J. *40*, e107405. https://doi.org/10.15252/embj.2020107405.

Cerutti, G., Guo, Y., Zhou, T., Gorman, J., Lee, M., Rapp, M., Reddem, E.R., Yu, J., Bahna, F., Bimela, J., et al. (2021). Potent SARS-CoV-2 neutralizing antibodies directed against spike N-terminal domain target a single supersite. Cell Host Microbe *29*, 819–833.e7. https://doi.org/10.1016/j.chom.2021.03.005.

Cerutti, G., Guo, Y., Liu, L., Liu, L., Zhang, Z., Luo, Y., Huang, Y., Wang, H.H., Ho, D.D., Sheng, Z., and Shapiro, L. (2022). Cryo-EM structure of the SARS-CoV-2 Omicron spike. Cell Rep. *38*, 110428. https://doi.org/10.1016/j.celrep.2022.110428.

Chi, X., Yan, R., Zhang, J., Zhang, G., Zhang, Y., Hao, M., Zhang, Z., Fan, P., Dong, Y., Yang, Y., et al. (2020). A neutralizing human antibody binds to the N-terminal domain of the Spike protein of SARS-CoV-2. Science *369*, 650–655. https://doi.org/10.1126/science.abc6952.

Collier, D.A., de Marco, A., Ferreira, I.A.T.M., Meng, B., Datir, R.P., Walls, A.C., Kemp, S.A., Bassi, J., Pinto, D., Silacci-Fregni, C., et al. (2021a). Sensitivity of SARS-CoV-2 B.1.1.7 to mRNA vaccine-elicited antibodies. Nature *593*, 136–141. https://doi.org/10.1038/s41586-021-03412-7.

Collier, D.A., Ferreira, I.A.T.M., Kotagiri, P., Datir, R.P., Lim, E.Y., Touizer, E., Meng, B., Abdullahi, A., CITIID-NIHR BioResource COVID-19 Collaboration; and Kingston, N., et al. (2021b). Age-related immune response heterogeneity to SARS-CoV-2 vaccine BNT162b2. Nature *596*, 417–422. https://doi.org/10.1038/s41586-021-03739-1.

Cui, Z., Liu, P., Wang, N., Wang, L., Fan, K., Zhu, Q., Wang, K., Chen, R., Feng, R., Jia, Z., et al. (2022). Structural and functional characterizations of infectivity and immune evasion of SARS-CoV-2 Omicron. Cell *185*, 860–871.e13. https://doi.org/10.1016/j.cell.2022.01.019.

Daniloski, Z., Jordan, T.X., Ilmain, J.K., Guo, X., Bhabha, G., Tenoever, B.R., and Sanjana, N.E. (2021). The spike d614g mutation increases sars-cov-2 infection of multiple human cell types. Elife *10*, e65365–16. https://doi.org/10.7554/eLife.65365.

Dhar, M.S., Marwal, R., VS, R., Ponnusamy, K., Jolly, B., Bhoyar, R.C., Sardana, V., Naushin, S., Rophina, M., Mellan, T.A., et al. (2021). Genomic characterization and epidemiology of an emerging SARS-CoV-2 variant in Delhi, India. Science *374*, 995–999. https://doi.org/10.1126/science.abj9932.

Díaz-Salinas, M.A., Li, Q., Ejemel, M., Yurkovetskiy, L., Luban, J., Shen, K., Wang, Y., and Munro, J.B. (2022). Conformational dynamics and allosteric

modulation of the SARS-CoV-2 spike. Elife *11*. https://doi.org/10.7554/eLife.75433.

Ferreira, I.A.T.M., Kemp, S.A., Datir, R., Saito, A., Meng, B., Rakshit, P., Takaori-Kondo, A., Kosugi, Y., Uriu, K., Kimura, I., et al. (2021). SARS-CoV-2 B.1.617 mutations L452R and E484Q are not synergistic for antibody evasion. J. Infect. Dis. *224*, 989–994. https://doi.org/10.1093/infdis/jiab368.

Gobeil, S.M.-C., Janowska, K., McDowell, S., Mansouri, K., Parks, R., Manne, K., Stalls, V., Kopp, M.F., Henderson, R., Edwards, R.J., et al. (2021). D614G mutation alters SARS-CoV-2 spike conformation and enhances protease cleavage at the S1/S2 junction. Cell Rep. *34*, 108630. https://doi.org/10.1016/j.celrep.2020.108630.

Gobeil, S.M.-C., Henderson, R., Stalls, V., Janowska, K., Huang, X., May, A., Speakman, M., Beaudoin, E., Manne, K., Li, D., et al. (2022). Structural diversity of the SARS-CoV-2 Omicron spike. Mol. Cell. *82*, 2050–2068.e6. https://doi.org/10.1016/j.molcel.2022.03.028.

Hoffmann, M., Kleine-Weber, H., and Pöhlmann, S. (2020a). A multibasic cleavage site in the spike protein of SARS-CoV-2 is essential for infection of human lung cells. Mol. Cell. *78*, 779–784.e5. https://doi.org/10.1016/j.molcel.2020.04.022.

Hoffmann, M., Kleine-Weber, H., Schroeder, S., Krüger, N., Herrler, T., Erichsen, S., Schiergens, T.S., Herrler, G., Wu, N.-H., Nitsche, A., et al. (2020b). SARS-CoV-2 cell entry depends on ACE2 and TMPRSS2 and is blocked by a clinically proven protease inhibitor. Cell *181*, 271–280.e8. https://doi.org/10.1016/j.cell.2020.02.052.

Jackson, C.B., Farzan, M., Chen, B., and Choe, H. (2022). Mechanisms of SARS-CoV-2 entry into cells. Nat. Rev. Mol. Cell. Biol. *23*, 3–20. https://doi.org/10.1038/s41580-021-00418-x.

Jaimes, J.A., Millet, J.K., and Whittaker, G.R. (2020). Proteolytic cleavage of the SARS-CoV-2 spike protein and the role of the novel S1/S2 site. iScience *23*, 101212. https://doi.org/10.1016/j.isci.2020.101212.

Johnson, B.A., Xie, X., Bailey, A.L., Kalveram, B., Lokugamage, K.G., Muruato, A., Zou, J., Zhang, X., Juelich, T., Smith, J.K., et al. (2021). Loss of furin cleavage site attenuates SARS-CoV-2 pathogenesis. Nature *591*, 293–299. https://doi.org/10.1038/s41586-021-03237-4.

Kemp, S.A., Collier, D.A., Datir, R.P., Ferreira, I.A.T.M., Gayed, S., Jahun, A., Hosmillo, M., Rees-Spear, C., Mlcochova, P., Lumb, I.U., et al. (2021). SARS-CoV-2 evolution during treatment of chronic infection. Nature *592*, 277–282. https://doi.org/10.1038/s41586-021-03291-y.

Kimura, I., Yamasoba, D., Nasser, H., Zahradnik, J., Kosugi, Y., Wu, J., Nagata, K., Uriu, K., Tanaka, Y.L., Ito, J., et al. (2022). SARS-CoV-2 spike S375F mutation characterizes the Omicron BA.1 variant. biorxiv. https://doi.org/10.1101/2022.04.03.486864.

Lempp, F.A., Soriaga, L.B., Montiel-Ruiz, M., Benigni, F., Noack, J., Park, Y.-J., Bianchi, S., Walls, A.C., Bowen, J.E., Zhou, J., et al. (2021). Lectins enhance SARS-CoV-2 infection and influence neutralizing antibodies. Nature *598*, 342–347. https://doi.org/10.1038/s41586-021-03925-1.

Li, D., Edwards, R.J., Manne, K., Martinez, D.R., Schäfer, A., Alam, S.M., Wiehe, K., Lu, X., Parks, R., Sutherland, L.L., et al. (2021). In vitro and in vivo functions of SARS-CoV-2 infection-enhancing and neutralizing antibodies. Cell *184*, 4203–4219.e32. https://doi.org/10.1016/j.cell.2021.06.021.

Liu, Y., Soh, W.T., Kishikawa, J.I., Hirose, M., Nakayama, E.E., Li, S., Sasai, M., Suzuki, T., Tada, A., Arakawa, A., et al. (2021). An infectivity-enhancing site on the SARS-CoV-2 spike protein targeted by antibodies. Cell *184*, 3452–3466.e18. https://doi.org/10.1016/j.cell.2021.05.032.

Liu, Y., Liu, J., Plante, K.S., Plante, J.A., Xie, X., Zhang, X., Ku, Z., An, Z., Scharton, D., Schindewolf, C., et al. (2022). The N501Y spike substitution enhances SARS-CoV-2 infection and transmission. Nature *602*, 294–299. https://doi.org/10.1038/s41586-021-04245-0.

Madu, I.G., Roth, S.L., Belouzard, S., and Whittaker, G.R. (2009). Characterization of a highly conserved domain within the severe acute respiratory syndrome coronavirus spike protein S2 domain with characteristics of a viral fusion peptide. J. Virol. *83*, 7411–7421. https://doi.org/10.1128/JVI.00079-09.

Mannar, D., Saville, J.W., Zhu, X., Srivastava, S.S., Berezuk, A.M., Tuttle, K.S., Marquez, A.C., Sekirov, I., and Subramaniam, S. (2022). SARS-CoV-2 Omicron variant: antibody evasion and cryo-EM structure of spike protein–ACE2 complex. Science *375*, 760–764. https://doi.org/10.1126/science.abn7760.

McCallum, M., Walls, A.C., Sprouse, K.R., Bowen, J.E., Rosen, L.E., Dang, H.v., de Marco, A., Franko, N., Tilles, S.W., Logue, J., et al. (2021). Molecular basis of immune evasion by the Delta and Kappa SARS-CoV-2 variants. Science *374*, 1621–1626. https://doi.org/10.1126/science.abl8506.

McCallum, M., de Marco, A., Lempp, F.A., Tortorici, M.A., Pinto, D., Walls, A.C., Beltramello, M., Chen, A., Liu, Z., Zatta, F., et al. (2021b). N-terminal domain antigenic mapping reveals a site of vulnerability for SARS-CoV-2. Cell *184*, 2332–2347.e16. https://doi.org/10.1016/j.cell.2021.03.028.

McCallum, M., Czudnochowski, N., Rosen, L.E., Zepeda, S.K., Bowen, J.E., Walls, A.C., Hauser, K., Joshi, A., Stewart, C., Dillen, J.R., et al. (2022). Structural basis of SARS-CoV-2 Omicron immune evasion and receptor engagement. Science *375*, 864–868. https://doi.org/10.1126/science.abn8652.

McCarthy, K.R., Rennick, L.J., Nambulli, S., Robinson-McCarthy, L.R., Bain, W.G., Haidar, G., and Duprex, W.P. (2021). Recurrent deletions in the SARS-CoV-2 spike glycoprotein drive antibody escape. Science *371*, 1139–1142. https://doi.org/10.1126/science.abf6950.

Meng, B., Kemp, S.A., Papa, G., Datir, R., Ferreira, I.A.T.M., Marelli, S., Harvey, W.T., Lytras, S., Mohamed, A., Gallo, G., et al. (2021). Recurrent emergence of SARS-CoV-2 spike deletion H69/V70 and its role in the Alpha variant B.1.1.7. Cell Rep. *35*, 109292. https://doi.org/10.1016/j.celrep.2021.109292.

Meng, B., Abdullahi, A., Ferreira, I.A.T.M., Goonawardane, N., Saito, A., Kimura, I., Yamasoba, D., Gerber, P.P., Fatihi, S., Rathore, S., et al. (2022). Altered TMPRSS2 usage by SARS-CoV-2 Omicron impacts infectivity and fusogenicity. Nature *603*, 706–714. https://doi.org/10.1038/s41586-022-04474-x.

Mishra, T., Dalavi, R., Joshi, G., Kumar, A., Pandey, P., Shukla, S., Mishra, R.K., and Chande, A. (2022). SARS-CoV-2 spike E156G/Δ157-158 mutations contribute to increased infectivity and immune escape. Life Sci. Alliance *5*, e202201415. https://doi.org/10.26508/lsa.202201415.

Mlcochova, P., Collier, D., Ritchie, A., Assennato, S.M., Hosmillo, M., Goel, N., Meng, B., Chatterjee, K., Mendoza, V., Temperton, N., et al. (2020). Combined point-of-care nucleic acid and antibody testing for SARS-CoV-2 following emergence of D614G spike variant. Cell Rep. Med. *1*, 100099. https://doi.org/10.1016/j.xcrm.2020.100099.

Mlcochova, P., Kemp, S., Dhar, M.S., Papa, G., Meng, B., Ferreira, I.A.T.M., Datir, R., Collier, D.A., Albecka, A., Singh, S., et al. (2021). SARS-CoV-2 B.1.617.2 Delta variant replication and immune evasion. Nature. https://doi.org/10.1038/s41586-021-03944-y.

Ou, T., Mou, H., Zhang, L., Ojha, A., Choe, H., and Farzan, M. (2021). Hydroxychloroquine-mediated inhibition of SARS-CoV-2 entry is attenuated by TMPRSS2. PLoS Pathog. *17*, e1009212. https://doi.org/10.1371/journal.ppat.1009212.

Papa, G., Mallery, D.L., Albecka, A., Welch, L.G., Cattin-Ortolá, J., Luptak, J., Paul, D., McMahon, H.T., Goodfellow, I.G., Carter, A., et al. (2021). Furin cleavage of SARS-CoV-2 Spike promotes but is not essential for infection and cell-cell fusion. PLoS Pathog. *17*, e1009246. https://doi.org/10.1371/journal.ppat.1009246.

Park, J.E., Li, K., Barlan, A., Fehr, A.R., Perlman, S., McCray, P.B., and Gallagher, T. (2016). Proteolytic processing of middle east respiratory syndrome coronavirus spikes expands virus tropism. Proc. Natl. Acad. Sci. USA *113*, 12262–12267. https://doi.org/10.1073/pnas.1608147113.

Peacock, T.P., Brown, J.C., Zhou, J., Thakur, N., Sukhova, K., Newman, J., Kugathasan, R., Yan, A.W.C., Furnon, W., De Lorenzo, G., et al. (2022). The altered entry pathway and antigenic distance of the SARS-CoV-2 Omicron variant map to separate domains of spike protein. biorxiv. https://doi.org/10.1101/2021.12.31.474653.

Peacock, T.P., Goldhill, D.H., Zhou, J., Baillon, L., Frise, R., Swann, O.C., Kugathasan, R., Penn, R., Brown, J.C., Sanchez-David, R.Y., et al. (2021). The furin cleavage site in the SARS-CoV-2 spike protein is required for

transmission in ferrets. Nat. Microbiol. *6*, 899–909. https://doi.org/10.1038/s41564-021-00908-w.

Peng, R., Wu, L.-A., Wang, Q., Qi, J., and Gao, G.F. (2021). Cell entry by SARS-CoV-2. Trends. Biochem. Sci. *46*, 848–860. https://doi.org/10.1016/j.tibs.2021.06.001.

Pizzato, M., Erlwein, O., Bonsall, D., Kaye, S., Muir, D., and McClure, M.O. (2009). A one-step SYBR Green I-based product-enhanced reverse transcriptase assay for the quantitation of retroviruses in cell culture supernatants. J. Virol. Methods. *156*, 1–7. https://doi.org/10.1016/j.jviromet.2008.10.012.

Qing, E., Kicmal, T., Kumar, B., Hawkins, G.M., Timm, E., Perlman, S., and Gallagher, T. (2021). Dynamics of sars-cov-2 spike proteins in cell entry: control elements in the amino-terminal domains. mBio *12*, e0159021. https://doi.org/10.1128/mBio.01590-21.

Qing, E., Li, P., Cooper, L., Schulz, S., Jäck, H.M., Rong, L., Perlman, S., and Gallagher, T. (2022). Inter-domain communication in SARS-CoV-2 spike proteins controls protease-triggered cell entry. Cell Rep. *39*, 110786. https://doi.org/10.1016/j.celrep.2022.110786.

Raghuvamsi, P. v., Tulsian, N.K., Samsudin, F., Qian, X., Purushotorman, K., Yue, G., Kozma, M.M., Hwa, W.Y., Lescar, J., Bond, P.J., et al. (2021). SARS-CoV-2 S protein:ACE2 interaction reveals novel allosteric targets. Elife *10*, e63646. https://doi.org/10.7554/eLife.63646.

Rajah, M.M., Hubert, M., Bishop, E., Saunders, N., Robinot, R., Grzelak, L., Planas, D., Dufloo, J., Gellenoncourt, S., Bongers, A., et al. (2021). SARS-CoV-2 Alpha, Beta, and Delta variants display enhanced Spike-mediated syncytia formation. EMBO J. *40*, e108944. https://doi.org/10.15252/embj.2021108944.

Ramanathan, M., Ferguson, I.D., Miao, W., and Khavari, P.A. (2021). SARS-CoV-2 B.1.1.7 and B.1.351 spike variants bind human ACE2 with increased affinity. Lancet. Infect. Dis. *21*, 1070. https://doi.org/10.1016/S1473-3099(21)00262-0.

Saville, J.W., Mannar, D., Zhu, X., Srivastava, S.S., Berezuk, A.M., Demers, J.-P., Zhou, S., Tuttle, K.S., Sekirov, I., Kim, A., et al. (2022). Structural and biochemical rationale for enhanced spike protein fitness in delta and kappa SARS-CoV-2 variants. Nat. Commun. *13*, 742. https://doi.org/10.1038/s41467-022-28324-6.

Simon-Loriere, E., and Schwartz, O. (2022). Towards SARS-CoV-2 serotypes? Nat. Rev. Microbiol. *20*, 187–188. https://doi.org/10.1038/s41579-022-00708-x.

Stalls, V., Lindenberger, J., Gobeil, S.M.-C., Henderson, R., Parks, R., Barr, M., Deyton, M., Martin, M., Janowska, K., Huang, X., et al. (2022). Cryo-EM structures of SARS-CoV-2 Omicron BA.2 spike. Cell Rep. *39*. https://doi.org/10.1016/j.celrep.2022.111009.

Supasa, P., Zhou, D., Dejnirattisai, W., Liu, C., Mentzer, A.J., Ginn, H.M., Zhao, Y., Duyvesteyn, H.M.E., Nutalai, R., Tuekprakhon, A., et al. (2021). Reduced neutralization of SARS-CoV-2 B.1.1.7 variant by convalescent and vaccine sera. Cell *184*, 2201–2211.e7. https://doi.org/10.1016/j.cell.2021.02.033.

Suryadevara, N., Shrihari, S., Gilchuk, P., VanBlargan, L.A., Binshtein, E., Zost, S.J., Nargi, R.S., Sutton, R.E., Winkler, E.S., Chen, E.C., et al. (2021). Neutralizing and protective human monoclonal antibodies recognizing the N-terminal domain of the SARS-CoV-2 spike protein. Cell *184*, 2316–2331.e15. https://doi.org/10.1016/j.cell.2021.03.029.

Suzuki, R., Yamasoba, D., Kimura, I., Wang, L., Kishimoto, M., Ito, J., Morioka, Y., Nao, N., Nasser, H., Uriu, K., et al. (2022). Attenuated fusogenicity and pathogenicity of SARS-CoV-2 Omicron variant. Nature *603*, 700–705. https://doi.org/10.1038/s41586-022-04462-1.

Ulrich, L., Halwe, N.J., Taddeo, A., Ebert, N., Schön, J., Devisme, C., Trüeb, B.S., Hoffmann, B., Wider, M., Fan, X., et al. (2022). Enhanced fitness of SARS-CoV-2 variant of concern Alpha but not Beta. Nature *602*, 307–313. https://doi.org/10.1038/s41586-021-04342-0.

V'kovski, P., Kratzel, A., Steiner, S., Stalder, H., and Thiel, V. (2021). Coronavirus biology and replication: implications for SARS-CoV-2. Nat. Rev. Microbiol. *19*, 155–170. https://doi.org/10.1038/s41579-020-00468-6.

Whittaker, G.R., Daniel, S., and Millet, J.K. (2021). Coronavirus entry: how we arrived at SARS-CoV-2. Curr. Opin. Virol. *47*, 113–120. https://doi.org/10.1016/j.coviro.2021.02.006.

Willett, B.J., Grove, J., MacLean, O.A., Wilkie, C., Logan, N., De Lorenzo, G., Furnon, W., Scott, S., Manali, M., Szemiel, A., et al. (2022). The hyper-transmissible SARS-CoV-2 Omicron variant exhibits significant antigenic change, vaccine escape and a switch in cell entry mechanism. medrxiv. https://doi.org/10.1101/2022.01.03.21268111.

Yamasoba, D., Kimura, I., Nasser, H., Morioka, Y., Nao, N., Ito, J., Uriu, K., Tsuda, M., Zahradnik, J., Shirakawa, K., et al. (2022). Virological characteristics of the SARS-CoV-2 omicron BA.2 spike. Cell. https://doi.org/10.1016/j.cell.2022.04.035.

Yang, Z., Han, Y., Ding, S., Shi, W., Zhou, T., Finzi, A., Kwong, P.D., Mothes, W., and Lu, M. (2022). SARS-CoV-2 variants increase kinetic stability of open spike conformations as an evolutionary strategy. mBio *13*, e0322721. https://doi.org/10.1128/mbio.03227-21.

Ye, G., Liu, B., and Li, F. (2022). Cryo-EM structure of a SARS-CoV-2 omicron spike protein ectodomain. Nat. Commun. *13*, 1214. https://doi.org/10.1038/s41467-022-28882-9.

Youk, J., Kim, T., Evans, K.v., Jeong, Y.-I., Hur, Y., Hong, S.P., Kim, J.H., Yi, K., Kim, S.Y., Na, K.J., et al. (2020). Three-dimensional human alveolar stem cell culture models reveal infection response to SARS-CoV-2. Cell Stem Cell *27*, 905–919.e10. https://doi.org/10.1016/j.stem.2020.10.004.

Yurkovetskiy, L., Wang, X., Pascal, K.E., Tomkins-Tinch, C., Nyalile, T.P., Wang, Y., Baum, A., Diehl, W.E., Dauphin, A., Carbone, C., et al. (2020). Structural and functional analysis of the D614G SARS-CoV-2 spike protein variant. Cell *183*, 739–751.e8. https://doi.org/10.1016/j.cell.2020.09.032.

Zhang, J., Xiao, T., Cai, Y., Lavine, C.L., Peng, H., Zhu, H., Anand, K., Tong, P., Gautam, A., Mayer, M.L., et al. (2021). Membrane fusion and immune evasion by the spike protein of SARS-CoV-2 Delta variant. Science *374*, 1353–1360. https://doi.org/10.1126/science.abl9463.

Zhang, J., Cai, Y., Xiao, T., Lu, J., Peng, H., Sterling, S.M., Walsh, R.M., Rits-Volloch, S., Zhu, H., Woosley, A.N., et al. (2021). Structural impact on SARS-CoV-2 spike protein by D614G substitution. Science *372*, 525–530. https://doi.org/10.1126/science.abf2303.

Zhang, J., Cai, Y., Lavine, C.L., Peng, H., Zhu, H., Anand, K., Tong, P., Gautam, A., Mayer, M.L., Rits-Volloch, S., et al. (2022). Structural and functional impact by SARS-CoV-2 Omicron spike mutations. Cell Rep. *39*, 110729. https://doi.org/10.1016/j.celrep.2022.110729.

Zhang, L., Jackson, C.B., Mou, H., Ojha, A., Peng, H., Quinlan, B.D., Rangarajan, E.S., Pan, A., Vanderheiden, A., Suthar, M.S., et al. (2020). SARS-CoV-2 spike-protein D614G mutation increases virion spike density and infectivity. Nat. Commun. *11*, 6013. https://doi.org/10.1038/s41467-020-19808-4.

Zhou, T., Wang, L., Misasi, J., Pegu, A., Zhang, Y., Harris, D.R., Olia, A.S., Talana, C.A., Yang, E.S., Chen, M., et al. (2022). Structural basis for potent antibody neutralization of SARS-CoV-2 variants including B.1.1.529. Science, 376. https://doi.org/10.1126/science.abn8897.

STAR★METHODS

KEY RESOURCES TABLE

REAGENT or RESOURCE	SOURCE	IDENTIFIER
Antibodies		
Anti-ACE2 antibody	R&D systems	Cat#AF933; RRID:AB_355722
Rabbit anti-SARS-CoV-2 S	Thermofisher	Cat#PA1-41165; RRID:AB_1087210
Mouse anti-SARS-CoV-2 S1	R&D systems	Cat#MAB105403
Mouse anti HIV-1 p55/p24	NIBSC	Cat#ARP313
Rabbit anti-GAPDH	Proteintech	Cat#10494-1-AP; RRID:AB_2263076
Anti-rabbit HRP conjugate	Cell Signaling	Cat#7074; RRID:AB_2099233
Anti-mouse HRP conjugate	Cell Signaling	Cat#7076; RRID:AB_330924
Goat anti-Rabbit IgG Alexa Fluor 647	Thermofisher	Cat#A21244; RRID:AB_2535812
Bacterial and virus strains		
XL1-blue cells	Agilent	Cat#200249
Biological samples		
Airway organoids	Joo-Hyeon Lee	N/A
Human Sera	Collier et al. (2021a)	N/A
Chemicals, peptides, and recombinant proteins		
E64D	Tocris	Cat#4545
Camostat	Sigma-Aldrich	Cat#SML0057
Fugene HD Transfection Reagent	Promega	Cat#E2311
Fugene 6 Transfection Reagent	Promega	Cat#E2691
Critical commercial assays		
Bright-Glo	Promega	Cat#E2650
QuikChange Lightning	Agilent	Cat#210518
QuantiTect SYBR Green PCR Kit	Qiagen	Cat#204143
Experimental models: Cell lines		
HEK293T	ATCC	Cat#CRL-3216
HEK293T-TMPRSS2	Leo James	N/A
HEK293T-ACE2ΔTMPRSS2	Leo James	N/A
HEK293T-GFP11	Leo James	N/A
Vero-GFP1-10	Leo James	N/A
Vero-ACE2/TMPRSS2	Emma Thomson	N/A
Calu3	Paul Lehner	N/A
A549-ACE2/TMPRSS2	Massimo Palmarini	N/A
NCI-H1299	Simon Cook	N/A
HeLa-ACE2	James Voss	N/A
Oligonucleotides		
SARS-CoV-2_Delta_G156E_Fwd: AG CTGGATGGAAAGCGAGGTGTACAG CAGCGCCAACAACTG	This paper	N/A
SARS-CoV-2_Delta_G156E_Rev: GC AGTTGTTGGCGCTGCTGTACACCT CGCTTTCCATCCAGCT	This paper	N/A
SARS-CoV-2_Delta_D142G_Fwd: G CAACGACCCCTTCCTGGGCGTCTA CTACCACAAGAAC	This paper	N/A

(Continued on next page)

Continued

REAGENT or RESOURCE	SOURCE	IDENTIFIER
SARS-CoV-2_Delta_D142G_Rev: GTTCTTGTGGTAGTAGACGCCCAGGAAGGGGTCGTTGC	This paper	N/A
SARS-CoV-2_Delta_157F158R_Fwd: GCTGGATGGAAAGCGGGTTCCGGGTGTACAGCAGCGCC	This paper	N/A
SARS-CoV-2_Delta_157F158R_Rev: GGCGCTGCTGTACACCCGGAACCCGCTTTCCATCCAGC	This paper	N/A
Recombinant DNA		
Plasmid: p8.91	Kemp et al. (2021)	N/A
Plasmid: pCSFLW	Kemp et al. (2021)	N/A
Plasmid: pcDNA-SARS-CoV-2-D614G-S-Δ19 WT	Meng et al. (2021); Mlcochova et al. (2021)	N/A
Plasmid: pcDNA-SARS-CoV-2-D614G-S-Δ19 ΔH69V70	Meng et al. (2021)	N/A
Plasmid: pcDNA-SARS-CoV-2-D614G-S-Δ19 Delta	Mlcochova et al. (2021)	N/A
Plasmid: pcDNA-SARS-CoV-2-D614G-S-Δ19 Kappa	Mlcochova et al. (2021)	N/A
Plasmid: pcDNA-SARS-CoV-2-D614G-S-Δ19 BA.1	This paper	N/A
Plasmid: pcDNA-SARS-CoV-2-D614G-S-Δ19 BA.2	This paper	N/A
Plasmid: pcDNA-SARS-CoV-2-D614G-S-Δ19 Delta + KappaNTD	This paper	N/A
Plasmid: pcDNA-SARS-CoV-2-D614G-S-Δ19 Kappa + DeltaNTD	This paper	N/A
Plasmid: pcDNA-SARS-CoV-2-D614G-S-Δ19 WT + DeltaNTD	This paper	N/A
Plasmid: pcDNA-SARS-CoV-2-D614G-S-Δ19 WT + KappaNTD	This paper	N/A
Plasmid: pcDNA-SARS-CoV-2-D614G-S-Δ19 BA.1 + DeltaNTD	This paper	N/A
Plasmid: pcDNA-SARS-CoV-2-D614G-S-Δ19 BA.2 + DeltaNTD	This paper	N/A
Plasmid: pcDNA-SARS-CoV-2-D614G-S-Δ19 BA.1 + BA.2NTD	This paper	N/A
Plasmid: pcDNA-SARS-CoV-2-D614G-S-Δ19 BA.2 + BA.1NTD	This paper	N/A
Plasmid: pcDNA-SARS-CoV-2-D614G-S-Δ19 Delta+681H/P	This paper	N/A
Plasmid: pcDNA-SARS-CoV-2-D614G-S-Δ19 Delta + KappaNTD+681H/P	This paper	N/A
Plasmid: pcDNA-SARS-CoV-2-D614G-S-Δ19 Delta D142G	This paper	N/A
Plasmid: pcDNA-SARS-CoV-2-D614G-S-Δ19 Delta G156E	This paper	N/A
Plasmid: pcDNA-SARS-CoV-2-D614G-S-Δ19 Delta 157F158R	This paper	N/A
Plasmid: pcDNA-SARS-CoV-2-D614G-S-Δ19 KappaRBD	Ferreira et al. (2021)	N/A

(*Continued on next page*)

CellPress
OPEN ACCESS

Continued

REAGENT or RESOURCE	SOURCE	IDENTIFIER
Software and algorithms		
GraphPad Prism	GraphPad Software	Version 9.4.0
FlowJo10	FlowJo	https://www.flowjo.com/solutions/flowjo/downloads
ImageJ	NIH	https://imagej.nih.gov/ij/
Other		
RNA (MS2)	Roche	Cat#10165948001
HIV RT	Milipore	Cat#382129
HindIII	NEB	Cat#R0104S
NheI	NEB	Cat#R3131S
Bsu36I	NEB	Cat#0524S

RESOURCE AVAILABILITY

Lead contact

Further information should be directed to and will be fulfilled by the Lead Contact, Bo Meng bm432@cam.ac.uk.

Materials availability

All unique reagents generated in this study are available from the lead contact.

Data and code availability

Raw data in this study is available from the lead contact upon request. This paper does not report original code. Any additional information required to reanalyze the data reported in this paper is available from the lead contact upon request.

EXPERIMENTAL MODEL AND SUBJECT DETAILS

The study was primarily a laboratory based study using pseudotyped virus (PV) with mutations generated by site-directed mutagenesis. We tested infectivity in a variety of model cell lines with drug inhibitors. Sensitivity to antibodies in serum was tested using sera collected from BNT162b2 vaccinees as part of the Cambridge NIHR Bioresource.

Ethical approval

Ethical approval for use of serum samples. Controls with COVID-19 were enrolled to the NIHR Bioresource Center Cambridge under ethics review board (17/EE/0025).

Cell culture

Calu3 (a human lung epithelial cell line; a gift from Paul Lehner) cells were maintained in Eagle's minimum essential medium containing 10% FBS and 1% PS. Vero-ACE2/TMPRSS2 cells (a monkey epithelial cell line overexpressing ACE2 and TMPRSS2; a gift from Emma Thomson), HeLa-ACE2 (a human cervix epithelial cell line overexpressing ACE2; a gift from James Voss) and A549-ACE2/TMPRSS2 (a human lung epithelial cell line overexpressing ACE2 and TMPRSS2; a gift from Massimo Palmarini) were maintained in Dulbecco's modified Eagle's medium (DMEM) containing 10% FBS and 1% PS. NCI-H1299 (a human lung epithelial cell line; a gift from Simon Cook) cells were maintained in RPMI containing 10% FBS and 1% PS. 293T (CRL-3216; a human kidney epithelial cell line) and its derivative cell lines including 293T-ACE2ΔTMPRSS2, 293T-TMPRSS2 and 293T-GFP11 have been described previously (Papa et al., 2021). All the 293T cell lines as well as Vero-GFP1-10 were maintained in DMEM with 10% FBS and 1% PS. All cells were regularly tested and are mycoplasma free. Airway epithelial organoids were obtained and maintained as previously described (Meng et al., 2022; Youk et al., 2020). Human distal lung parenchymal tissues were obtained from adult donors with no background lung pathologies from Papworth Hospital Research Tissue Bank (T02233). Airway organoids were cultured in 48-well plate and were passaged every 2 weeks as previously reported (Meng et al., 2022).

METHOD DETAILS

Plasmids

pcDNA-SARS-CoV-2-D614G-S WT, Delta, Kappa, Omicron BA.1 and Omicron BA.2 plasmids with 19 amino acid deletion at the C-terminus were generated by gene synthesis. For the construction of the chimeras, the region encompassing the NTD was digested

with Bsu36I and HindIII or NheI (all NEB) before being gel purified and ligated back to the respective backbone that was cut with the same pair of restriction enzymes. Amino acid substitutions in the Delta NTD (D142G, G156E and repair of 157F and 158R) or 681H or 681P were introduced into the pcDNA-SARS-CoV-2-D614G-Delta-S plasmid using the QuikChange Lightning Site-Directed Mutagenesis kit, following the manufacturer's instructions (Agilent Technologies). Sequences were checked by Sanger sequencing. The constructs of Kappa RBD only, ΔH69V70, p8.91 HIV-1 gag-pol expression vector and pCSFLW were reported previously (Ferreira et al., 2021; Meng et al., 2021).

Pseudotype virus preparation and infectivity titration

Viral vectors were prepared by transfection of 293T cells using Fugene HD transfection reagent (Promega) as described previously (Kemp et al., 2021). In brief, a 10 cm dish of 293T cells were transfected with a mixture of 11 μL of Fugene HD, 1 μg of pcDNAΔ19 spike, 1 μg of p8.91 HIV-1 gag-pol expression vector and 1.5 μg of pCSFLW (expressing the firefly luciferase reporter gene with the HIV-1 packaging signal). Viral supernatant was collected at 48 h after transfection, filtered through 0.45 μm filter or clarified through centrifugation and stored at −80°C. Infectivity was measured by luciferase detection (Bright Glo; Promega) in target cells. The raw readings (in relative light unit (RLU)) were then normalised with the SG-PERT and plotted using GraphPad Prism 9.

PV SG-PERT

The SARS-CoV-2 spike-pseudotyped viruses containing supernatants were standardised using an SYBR Green-based product-enhanced PCR assay (SG-PERT) as described previously (Pizzato et al., 2009). Briefly, 10-fold dilutions of virus supernatant were lysed in a 1:1 ratio in a 2x lysis solution (made up of 40% glycerol v/v 0.25% Triton X-100 v/v 100mM KCl, RNase inhibitor 0.8 U/mL, TrisHCL 100mM, buffered to pH7.4) for 10 min at room temperature. Sample lysates (12 μL) were added to 13 μL of SYBR Green master mix (containing 0.5 μM of MS2-RNA Fwd and Rev primers, 3.5 pmol/mL of MS2-RNA, and 0.125 U/μL of Ribolock RNAse inhibitor) and cycled in a QuantStudio (Thermofisher). Relative amounts of reverse transcriptase activity were determined as the rate of transcription of bacteriophage MS2 RNA, with absolute RT activity calculated by comparing the relative amounts of RT to an RT standard of known activity.

Western blot

For cell lysates, transfected 293 cells were washed and lysed in cell lysis buffer (Cell Signaling). Lysates were then diluted with 4 × sample buffer (Biorad) and boiled for 10 min before subjected to western blotting. For virions, clarified supernatants were loaded onto a thin layer of 8.4% optiprep density gradient medium (Sigma-Aldrich) and placed in a TLA55 rotor (Beckman Coulter) for ultracentrifugation for 2 h at 20,000 rpm. The pellet was then resuspended for western blotting. For protein detection, the following antibodies were used: rabbit anti-SARS-CoV-2 S monoclonal antibody (Thermofisher), mouse anti-SARS-CoV-2 S1 (R&D systems), rabbit anti-GAPDH polyclonal antibody (Proteintech), horseradish peroxidase (HRP)-conjugated anti-rabbit and anti-mouse IgG polyclonal antibody (Cell Signaling). Chemiluminescence was detected using ChemiDoc Touch Imaging System (Bio-Rad). The cleavage ratio of S1 or S2 to FL was determined by densitometry using ImageJ (NIH).

Drug and receptor blocking assay

For drug assay, A549-ACE2-TMPRSS2 (A549-A2T2) cells or human airway organoids were either E64D (Tocris) or camostat (Sigma-Aldrich) treated for 2 h at each drug concentration. For receptor blocking assay, 293T-ACE2ΔTMPRSS2 cells were treated with a series titrations of anti-ACE2 antibody (R&D systems) for 2 h. This was then followed by the addition of a comparable amount of input viruses pseudotyped with Delta, Kappa or chimeras (approx. 100,000 RLU). The cells were then left for 48 h before addition of substrate for luciferase (Promega) and read on a Glomax plate reader (Promega). The RLU was normalised against the non-drug or non-antibody control which was set as 100%.

Cell-cell fusion assay

Cell-cell fusion assays were described previously (Meng et al., 2022). Briefly, 293T GFP11 and Vero-GFP1-10 cells were seeded at 80% confluence in a 1:1 ratio in a 48 multiwell plate the day before.

Cells were co-transfected with 250 ng of spike expression plasmids using Fugene 6 following the manufacturer's instructions (Promega). Cell-cell fusion was measured using an Incucyte and determined as the proportion of green area to total phase area over time. Data were normalised to non-transfected control. Graphs were generated using GraphPad Prism 9.

FACS for surface spike expression

293T cells were seeded in a 24 multiwell plate the day before. On the following day 500 ng of spike expressors, together with 200 ng of pEF-eGFP expressor for transfection control, were transfected using Fugene 6 following the manufacturer's instructions (Promega). A day later, the cells were collected and washed for surface staining using 1 μL of rabbit anti-SARS-CoV-2 S polyclonal antibody (Thermofisher) per condition followed by secondary staining using goat anti-rabbit IgG Alexa 647 (Thermofisher) before analysing on LSR-II Pacman flow cytometer. A well of pEF-eGFP only transfected cells were served as a negative control.

CellPress
OPEN ACCESS

Neutralisation assay

This was performed as previously described (Collier et al., 2021a, 2021b).

QUANTIFICATION AND STATISTICAL ANALYSIS

Experiments were done at least two times with two to four technical replicates. Relative luciferase units were measured with a Glomax luminometer. Data were analyzed using GraphPad PRISM software (version 9.4.0). Statistical tests are described in the figure legends along n, mean, and standard deviation/error, where appropriate. Significant differences are annotated as *$p < 0.05$; **$p < 0.01$; ***$p < 0.001$; ****$p < 0.0001$.

Cell Reports, Volume *40*

Supplemental information

SARS-CoV-2 spike N-terminal domain modulates TMPRSS2-dependent viral entry and fusogenicity

Bo Meng, Rawlings Datir, Jinwook Choi, CITIID-NIHR Bioresource COVID-19 Collaboration, John R. Bradley, Kenneth G.C. Smith, Joo Hyeon Lee, and Ravindra K. Gupta

Figure S1

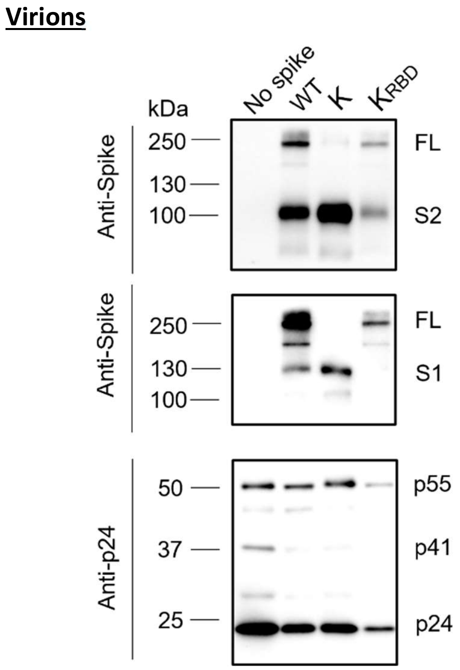

Supplementary Figure 1: Western blot of purified PV bearing RBD mutations only in SARS-CoV-2 Kappa and Kappa spike, together with WT and non-spike control. The sizes of protein markers were labelled to the left of the blot and the corresponding bands were labelled to the right. Related to Figure 1.

Figure S2

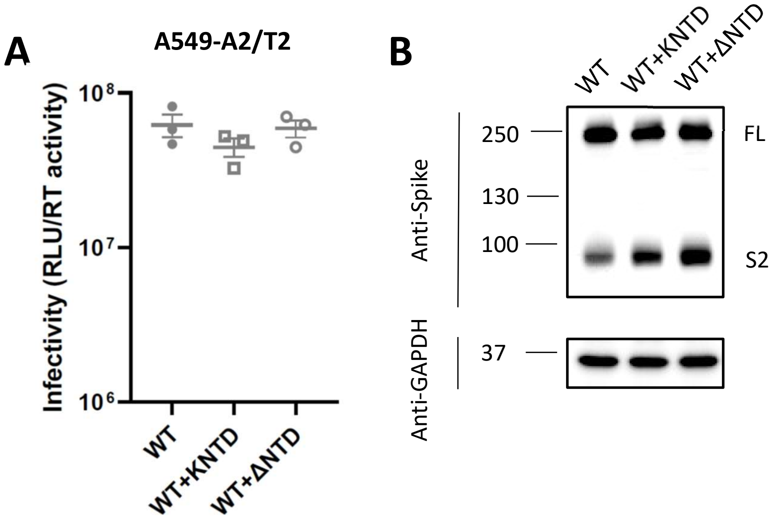

Supplementary Figure 2: The infectivity of SARS-CoV-2 WT, WT with Kappa NTD or Delta NTD chimeras in A549-A2/T2 cells (A) and western blot of 293T cell lysates showing spike cleavage (B). The sizes of protein markers were labelled to the left of the blot and the corresponding bands were labelled to the right. Related to Figure 1F.

Figure S3

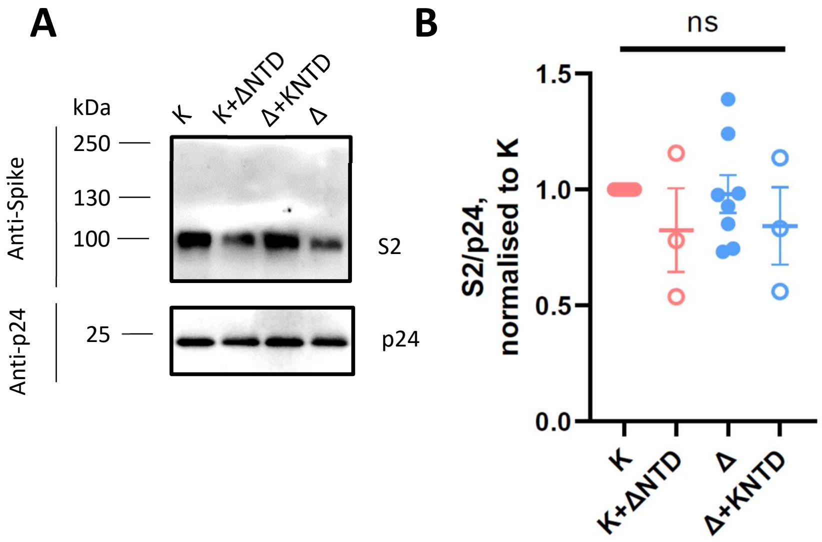

Supplementary Figure 3: (A): Western blots of purified PV bearing either SARS-CoV-2 Kappa, Delta or its chimeric spikes. The sizes of protein markers were labelled to the left of the blot and the corresponding bands were labelled to the right. (B): The intensity of the spike- and p24-associated bands on the western blots was densitometrically quantified (ImageJ) before the ratio was calculated for spike incorporation. Each dot represents one PV preparation and error bars represent SEM . The statistical analyses were done with one sample t test showing no significant difference. Related to Figure 2.

Figure S4

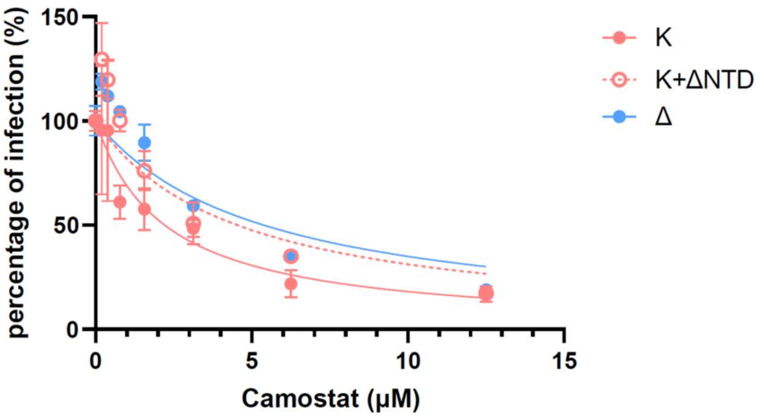

Supplementary Figure 4: The entry efficiency of SARS-CoV-2 Delta, Kappa and Kappa bearing the DeltaNTD in A549-ACE2/TMPRSS2 cells in the presence of TMPRSS2 specific inhibitor camostat. The RLU was normalised with non-drug control giving a percentage of infection. The data showing the SEM from 2 technical replicates; the error bars that lie within the datum points are not shown. Data are representative of two experiments. Related to Figure 2E.

Figure S5

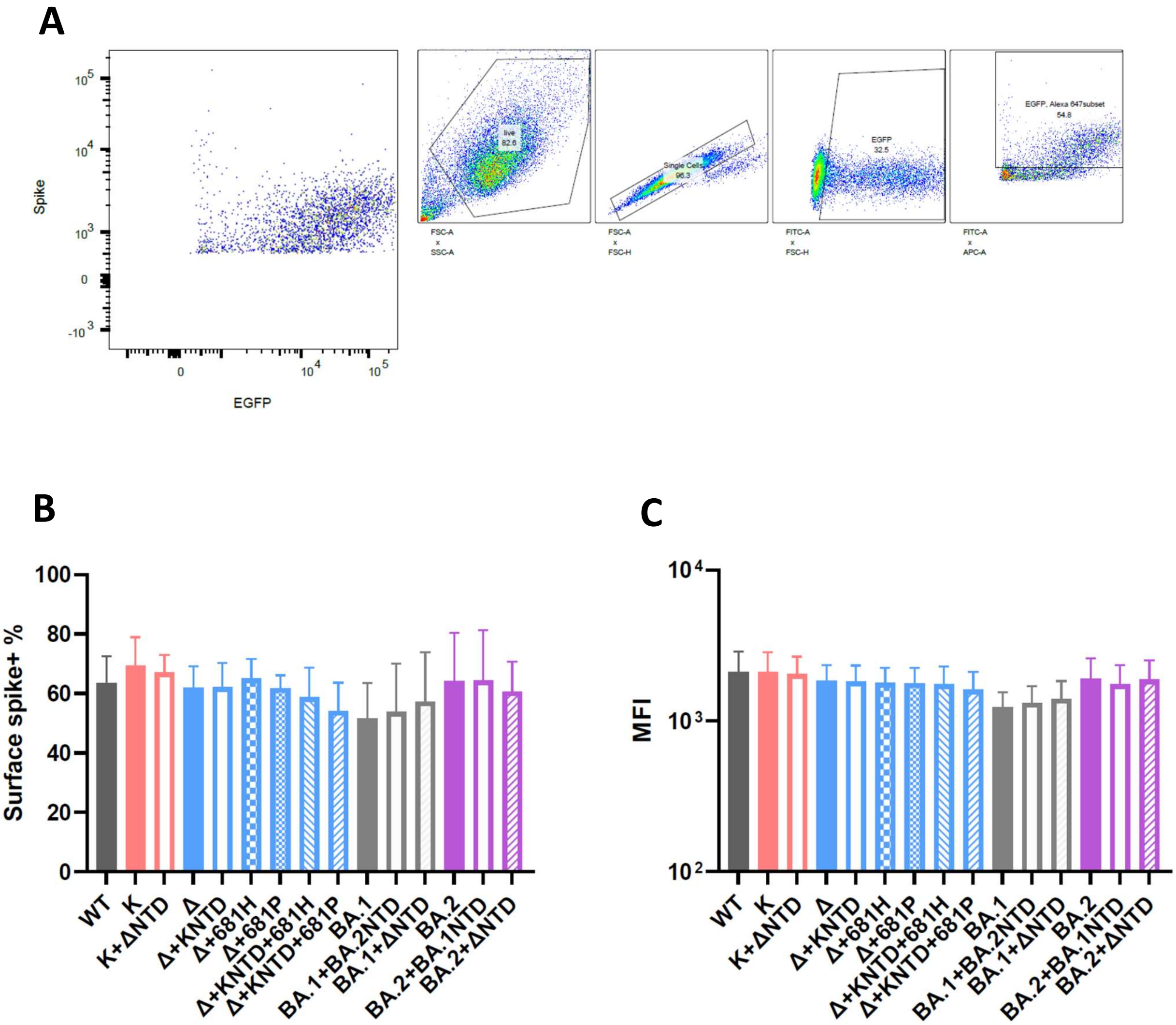

Supplementary Figure 5: (A): Gating strategy used to obtain the surface spike positive population in the transfected 293T cells. The spike positive cells from the eGFP positive cells were plotted either in percentage (B) or MFI (C) across different spikes. The error bars show the SEM from two experiments and the statistical analysis between each spike and WT was analysed with a paired t test, showing no significant difference. Related to Figures 4 and 5.
